# Development of Physical Fitness in Young Athletes: A Longitudinal Meta-analysis

**DOI:** 10.1186/s40798-026-01109-7

**Published:** 2026-09-29

**Authors:** Ahlan B. Lima, Ricardo T. Quinaud, Gabriel F. Reis, Fábio C. Karasiak, Eva Ageberg, Filipe M. Clemente, Carlos E. Gonçalves, Humberto M. Carvalho

**Affiliations:** 1 School of Sports, Federal University of Santa Catarina, Florianópolis, SC Brazil; 2 Department of Physical Education, University of the Extreme South of Santa Catarina, Criciúma, SC Brazil; 3 Graduate Program in Psychology, Federal University of Santa Catarina, Florianópolis, SC Brazil; 4 Department of Health Sciences, Faculty of Medicine, Lund University, PO Box 117, SE-221 00 Lund, Sweden; 5 Polytechnic University of Coimbra, Coimbra, Portugal; 6 Sport Physical Activity and Health Research & Innovation Center, Applied Research Institute, Polytechnic University of Coimbra, Coimbra, Portugal; 7 Department of Biomechanics and Sport Engineering, Gdansk University of Physical Education and Sport, Gdansk, Poland; 8 Faculty of Sport Sciences and Physical Education, University of Coimbra, Coimbra, Portugal

**Keywords:** Youth sports, Adolescent development, Athletic performance, Bayesian approach, Multilevel analysis

## Abstract

**Background:**

Understanding how physical fitness develops during adolescence is critical for interpreting and optimizing training and setting realistic performance expectations in youth sport. Yet, evidence-based, age-referenced benchmarks that track multiple fitness components across sports remain lacking, leaving practitioners without reliable normative trajectories to guide athlete development.

**Purpose:**

This longitudinal meta-analysis estimated developmental trajectories for vertical jump, sprint, and endurance performance in male youth athletes across multiple sports using data from longitudinal studies.

**Methods:**

Following PRISMA guidelines, we conducted a systematic search of MEDLINE, Web of Science, and Scopus for studies with repeated measurements of vertical jump, 10-meter sprint, and endurance performance in male youth athletes, from database inception to October 4, 2025. Analyses were restricted to males due to an insufficient number of eligible studies including female athletes. We applied a risk-of-bias assessment adapted from the Cochrane risk-of-bias tool and examined publication bias through funnel plot inspection and Egger’s regression test. We used Bayesian multilevel growth curve modeling to estimate age-related developmental trajectories, with analyses stratified by sport and test protocol.

**Results:**

Twenty-eight studies met eligibility criteria, providing 41 independent samples across nine sports. Vertical jump and endurance performance exhibited curvilinear trajectories with peak velocity occurring at 13.2 years (68% CrI [±1 posterior SD]: 13.0; 13.5) and 13.8 years (68% CrI: 13.6; 14.2), respectively, overlapping the typical period of peak height velocity in male athletes. Sprint performance showed linear improvement throughout adolescence (ages 12–17) without distinct acceleration periods. Sport-specific analyses revealed varying absolute values but broadly similar developmental patterns within each fitness component, with rugby exhibiting the highest vertical jump values and minimal sport differences observed for sprint development.

**Conclusion:**

The present age-referenced trajectories offer practical benchmarks for monitoring progression and setting realistic expectations in youth sport contexts. Our findings reveal distinct developmental patterns—curvilinear trajectories for vertical jump and endurance with peak improvements around ages 13–14 versus linear progression for sprint—with peak velocities coinciding with typical maturation timing

*Trial registration*: CRD42020175084 (PROSPERO) 25/03/2020.

**Supplementary Information:**

The online version contains supplementary material available at https://doi.org/10.1186/s40798-026-01109-7.

## Key Points

- Vertical jump and endurance performance follow curvilinear developmental trajectories, with peak velocities at ages 13.2 and 13.8 years, respectively–coinciding with typical peak height velocity timing in male youth athletes–while sprint performance develops linearly throughout adolescence, indicating that different fitness components require distinct monitoring and training approaches during youth development.
- Sport-specific developmental curves reveal substantial variation in absolute performance values (e.g., rugby showing highest vertical jump values, minimal sport differences in sprint), yet similar developmental patterns within each fitness component, supporting the use of sport-specific benchmarks for contextualized athlete monitoring.
- The age-referenced trajectories provided offer practical benchmarks for monitoring fitness progression across multiple sports, emphasizing that physical development reflects interacting factors of biological growth, training exposure, and sport-specific demands rather than chronological age alone.

**Supplementary Information:**

The online version contains supplementary material available at https://doi.org/10.1186/s40798-026-01109-7.

## Background

Physical fitness development in youth athletes is inherently multidimensional, encompassing neuromuscular power, speed, and aerobic capacity as fundamental components that underpin performance across diverse sporting contexts [1–3]. Vertical jump performance serves as a reliable indicator of explosive strength and lower-body power [1, 4], sprint performance reflects rapid acceleration and maximal velocity capabilities [5], and endurance capacity represents the ability to sustain high-intensity efforts and recover between repeated exercise bouts [6, 7]. These fitness components demonstrate distinct physiological underpinnings and developmental trajectories during adolescence [8]. Power and sprint performance are heavily influenced by neuromuscular maturation and muscle mass development, often showing pronounced improvements during peak height velocity (PHV) [9], while endurance capacity development is more closely linked to cardiovascular and metabolic maturation, following different temporal patterns [10]. Furthermore, the relative importance of these components varies across sports, with different disciplines emphasizing specific fitness domains [11]. Understanding the developmental trajectories of multiple fitness components and their interrelationships is fundamental to evidence-based long-term athlete development systems [12, 13].

Longitudinal study designs are essential for interpreting developmental patterns, as they enable tracking of intra-individual change over time across multiple fitness components while accounting for substantial inter-individual variability inherent in adolescent populations [14]. Such designs allow researchers to disentangle the relative contributions of chronological age, biological maturation, training exposure, and sport-specific demands on the development of different performance capacities [15, 16]. However, conducting rigorous longitudinal studies tracking multiple fitness components in applied youth sports contexts presents considerable challenges, including participant retention, standardization of measurement protocols over extended timeframes, accounting for maturation-related confounders, and substantial resource requirements [14]. Consequently, individual longitudinal studies are often limited by small sample sizes, restricted age ranges, limited fitness component coverage, and sport- or region-specific populations that constrain generalizability [17]. The fragmented nature of evidence across multiple sports, fitness components, and studies limits the development of comprehensive, evidence-based guidelines for youth sports programs [18].

Longitudinal meta-analysis offers a powerful approach to synthesizing repeated-measures data across multiple studies [17, 19], enabling researchers to aggregate individual developmental curves, estimate population-level growth parameters for different fitness domains, and examine moderating factors that may influence trajectories across performance indicators. Although there are repeated-measures studies examining physical fitness in youth athletes, systematic synthesis of repeated-measures data across multiple fitness components remains limited in sport science. Bayesian multilevel modeling provides the methodological framework for such synthesis [20, 21], accommodating the nested structure wherein repeated measurements are nested within individuals, individuals within studies, and studies within sports, while simultaneously modeling fitness outcomes. Bayesian statistical methods offer additional advantages [21, 22], including principled uncertainty quantification, natural accommodation of missing data, and probabilistic interpretation of results that facilitate direct comparisons of developmental patterns across sports and fitness components.

In this study, we estimated population-level developmental trajectories for three fitness components (vertical jump, sprint, and endurance performance) using available data in youth sports. Additionally, we quantified between-study and between-sport variation. This work addresses both a substantive gap regarding multidimensional fitness development in youth athletes and a methodological gap by illustrating an advanced yet underutilized analytical approach for synthesizing longitudinal observational data across heterogeneous sources.

## Methods

### Open Science Practices

Following the PRISMA guidelines [23] and recommendations on reproducibility in meta-analyses [24], we pre-registered the study protocol (PROSPERO: CRD42020175084) before conducting the searches. A repository containing the detailed methods, the full search strategy data, and the code necessary to recreate the analyses and figures in this study is available at https://osf.io/s6aeg/.

### Search Strategy

Two authors independently conducted the database searches and cross-checked results. We conducted an electronic search in the MEDLINE, Scopus, and Web of Science databases, covering the period from database inception to October 4, 2025, with no lower date limit applied. The search strategy combined terms related to: (i) youth or adolescent athletes; (ii) physical fitness indicators (vertical jump, countermovement jump, sprint, speed, endurance, aerobic capacity, Yo-Yo test); (iii) longitudinal design or repeated measures; and (iv) organized sports. Detailed methods and the full search strategy are summarized in the supplementary materials (https://osf.io/s6aeg/), following previous growth studies meta-analyses in young athletes [17, 25].

### Eligibility Criteria

Studies were included if they: (i) had a longitudinal design (minimum duration of 6 months), including both prospective and retrospective designs; (ii) reported at least three repeated measures of one or more physical fitness indicators—countermovement jump (CMJ) with or without arm swing, linear sprint performance (10 m), or intermittent/continuous endurance tests (e.g., Yo-Yo Intermittent Recovery Test, 20 m shuttle run)—between the ages of 8 and 17 years; (iii) involved young male athletes participating in organized individual or team sports (analyses were restricted to males due to an insufficient number of eligible studies including female athletes to support separate analyses); and (iv) were original articles published in English in peer-reviewed journals. The search strategy itself was not restricted by language of publication; the English-language restriction was applied only as an eligibility criterion at full-text review. Eligible studies required a longitudinal design with at least three repeated, standardized fitness measurements in organized youth sport contexts, characteristics typically associated with institutionally supported research conducted by university-affiliated groups and published in indexed, English-language sports science journals. Grey literature and non-English-language sources were therefore considered unlikely to contain a meaningful volume of additional eligible evidence meeting these criteria.

Studies were excluded if: (i) they included mixed-sex samples without separate data by sex; (ii) provided insufficient data for analysis (e.g., missing means, variability, or sample size at each measurement occasion), reported outcomes in incompatible formats (e.g., maximal oxygen consumption instead of distance covered for endurance tests), or lacked a comprehensive description of the sport, competitive level, or training environment; (iii) included athletes with known developmental disorders or medical conditions affecting growth. When studies appeared eligible but had incomplete reporting, authors were contacted to request missing data. Studies for which data could not be retrieved, either because authors did not respond or because the original data were no longer available (e.g., older publications), were excluded.

### Data Extraction

Two authors independently screened titles and abstracts based on eligibility criteria, using the *Rayyan* website [26]. We reviewed the full-text versions of selected studies, applying the inclusion criteria, and eligible studies were included in the meta-analysis. For each included study, we extracted: study characteristics (author, year, country, sample size, sport type, competitive level), participant characteristics (age at each measurement, biological maturation status when available), and physical fitness outcomes (mean and standard deviation or standard error for vertical jump height [cm], sprint time [s], and endurance performance [m]). We used the WebPlotDigitizer tool [27] to extract data from figures and plots when numerical data were not provided in tables. When multiple articles from the same cohort were identified, the most comprehensive article was selected to avoid duplication and ensure robust data synthesis. Disagreements between reviewers were resolved through discussion with a third author.

### Assessment of Risk of Bias and Publication Bias

We adapted the Cochrane risk-of-bias tool for randomized trials [28] to assess bias focusing on four domains: study design (adequate description of cohort, inclusion/exclusion criteria, and sport context), incomplete data or attrition (dropout rates, loss to follow-up, and handling of missing data), measurement of the outcome (standardized and validated measurement protocols for fitness indicators), and selective reporting (completeness of reporting across time points and outcomes). We formulated a checklist with specific questions for each domain, provided as supplementary material. Two reviewers independently assessed risk of bias for each study, with disagreements resolved through discussion.

We examined publication bias using a Bayesian Egger-type regression, in which standardized study-level effects (effect divided by standard error) were regressed on the inverse standard error, with a multilevel structure accounting for repeated observations within study. Because included studies contributed effects across a developmental age range rather than a single estimate per study, we additionally included an age-centered × inverse-standard-error interaction term to separate genuine developmental signal from precision-related asymmetry. The model intercept is the parameter conventionally interpreted as indicating small-study effects or publication bias; the slope on inverse standard error is not interpreted, consistent with standard practice in the meta-analysis literature. We additionally examined an alternative approach regressing individual-level model residuals against inverse sample size [29].

### Statistical Analysis

Throughout, we report 68% credible intervals (CrIs) defined as approximately ±1 posterior standard deviation (SD). This is motivated by the practical application of these data as age-referenced normative benchmarks for individual athlete monitoring, analogous to the ±1 SD bands used in standard growth references, where this band conventionally captures approximately two thirds of the expected distribution. These 68% intervals should not be interpreted as inferential CrIs for the population-level mean trajectory—90% CrIs for population-level parameters are narrower and are available in the online repository. Individual-level posterior predictive intervals, which incorporate residual within-study variance and are therefore wider than the 68% reference bands, are also available in the repository for practitioners requiring prediction intervals at the individual athlete level.

#### Model Specification

We fitted longitudinal meta-analysis models to describe the development of three physical fitness indicators (vertical jump, sprint, and endurance) in young athletes, using a fully Bayesian approach [17, 25]. We fitted separate multilevel polynomial growth models for each fitness indicator due to their distinct measurement scales and developmental trajectories. For each outcome $y_{ij}$ (e.g., vertical jump height) at time point t (chronological age) for individual i in study j, we assumed the within-study sampling errors were normally distributed.

To capture physical fitness development during adolescence, we fitted nonlinear multilevel models (polynomial functions) with growth coefficients up to the cubic term to adequately model the curvilinear patterns of development during puberty. We present outcome-specific model equations below, as the three outcomes differ in their polynomial degree and test-protocol structure. In all equations, β coefficients represent population-level effects, u coefficients represent group-level (study-level) effects allowing intercepts and growth coefficients to vary by study, and $\epsilon _{ij}$ represents residual error. Age was centered on the sample mean (approximately 14 years across outcomes) prior to all analyses, ensuring that the intercept describes expected performance at the mean observed age and reducing multicollinearity among polynomial terms. To facilitate model convergence, correlations between group-level intercepts and slopes were set to zero when computing the covariance matrix for vertical jump and endurance models; for the sprint model, correlations between group-level effects were retained and modeled via an LKJ(1) prior.

The model for vertical jump was specified as:

$$y_{ij} = \beta _0 + \beta _1 \cdot \text {age}_{ij} + \beta _2 \cdot \text {age}^2_{ij} + \beta _3 \cdot \text {age}^3_{ij} + \beta _{\text {test}} \cdot \text {test}_{ij} + u_{0j} + u_{1j} \cdot \text {age}_{ij} + u_{2j} \cdot \text {age}^2_{ij} + \epsilon _{ij},$$

where $\text {test}_{ij}$ is an indicator for CMJ without arm swing. A cubic polynomial was used as preliminary model comparison indicated it adequately captured the curvilinear developmental pattern. Group-level effects were specified up to the quadratic term ($u_{0j}, u_{1j} \cdot \text {age}_{ij}, u_{2j} \cdot \text {age}^2_{ij}$); a varying cubic slope was not included as it led to convergence difficulties given the number of available studies.

The model for 10-m sprint was specified as:

$$y_{ij} = \beta _0 + \beta _1 \cdot \text {age}_{ij} + \beta _2 \cdot \text {age}^2_{ij} + u_{0j} + u_{1j} \cdot \text {age}_{ij} + \epsilon _{ij}.$$

The cubic term was excluded based on preliminary model comparison and 10-fold cross-validation, which indicated equivalent predictive performance between the linear-quadratic and alternative specifications. No test-protocol moderator was required as analyses were restricted to 10-m sprint performance.

The model for endurance was specified as:

$$y_{ij} = \beta _0 + \beta _1 \cdot \text {age}_{ij} + \beta _2 \cdot \text {age}^2_{ij} + \beta _3 \cdot \text {age}^3_{ij} + \beta _{\text {test}} \cdot \text {test}_{ij} + u_{0j} + u_{1j} \cdot \text {age}_{ij} + u_{2j} \cdot \text {age}^2_{ij} + \epsilon _{ij},$$

where $\text {test}_{ij}$ takes values for the Interval Shuttle Run Test and Yo-Yo IR1 relative to the 20 m Shuttle Test as the reference category.

To examine between-sport heterogeneity, we included sport type as a categorical population-level moderator in extended models, allowing us to estimate sport-specific developmental trajectories and test for differential effects across sports.

Different testing protocols within each fitness component yield systematically different absolute values. For vertical jump, the CMJ with arm swing produces higher values than CMJ without arm swing due to the biomechanical contributions of upper-limb momentum; this was accommodated as a categorical test-type population-level effect ($\beta _{\text {test}}$) in the model. For endurance, the Yo-Yo IR1, 20 m Shuttle Test, and Interval Shuttle Run Test differ in whether effort is continuous or intermittent; these were modeled with separate intercept estimates while constraining the developmental trajectory shape to be common across protocols. Conversion between protocols was not performed, as conversion equations introduce additional uncertainty and are not universally applicable across sports and developmental stages. Cross-protocol comparisons in this study are therefore valid for the shape and timing of developmental trajectories but not for absolute performance values.

#### Prediction of the Age at Peak Velocity

We estimated the age at peak velocity (APV) of physical fitness development and the magnitude of peak velocity for each fitness indicator based on the first and second derivatives of the multilevel polynomial growth curve using the population-level parameters [30]. The first derivative identifies the maximum rate of change (peak velocity), while setting the second derivative to zero identifies the age at which this maximum occurs. For the sprint model (quadratic in age), APV was derived as: APV $= -\hat{\beta }_\text {age} / (2\hat{\beta }_{\text {age}^2}) + \bar{x}_\text {age}$. For the vertical jump and endurance models (cubic in age), APV was derived as: APV $= -(2\hat{\beta }_{\text {age}^2}) / (6\hat{\beta }_{\text {age}^3}) + \bar{x}_\text {age}$.

#### Priors

We specified weakly regularizing priors in our models to constrain parameter estimates to plausible ranges while allowing the data to dominate inference. Given the different measurement scales across fitness indicators, we tailored prior specifications to each outcome. Because age was centered on the sample mean (approximately 14 years; see Model specification), the intercept prior ($\beta _0$) describes expected performance at the mean observed age rather than at age zero, improving prior calibration relative to the raw age scale.

For the vertical jump model, we used: $\beta _0 \sim \text {Normal}(0, 10)$ for the intercept, $(\beta _1, \beta _2, \beta _3) \sim \text {Normal}(0, 5)$ for the age coefficients (linear, quadratic, and cubic terms), and $\beta _{\text {test}} \sim \text {Normal}(0, 5)$ for the test type effect (CMJ without arm swing). This intercept prior implies a 95% prior plausible range of approximately ±20 cm around the mean-age value, spanning the biologically plausible range for youth athletes; the age coefficient prior implies annual rates of change within ±10 cm/year, wider than empirically observed values (∼2–5 cm/year), confirming weak regularization. For group-level standard deviations, we specified $\sigma \sim \text {Exponential}(1)$ for study-level varying effects (intercept and age coefficients) and test-level varying effects.

For the sprint model, we used: $\beta _0 \sim \text {Normal}(0, 2)$ for the intercept and $(\beta _1, \beta _2) \sim \text {Normal}(0, 2)$ for the age coefficients (linear and quadratic terms only, as preliminary analyses indicated the cubic term was not necessary). This intercept prior implies a 95% range of approximately ±4 s around the mean-age value (observed: approximately 1.8–2.0 s), providing appropriate regularization. For group-level standard deviations, we specified $\sigma \sim \text {Exponential}(1)$. Unlike the other models, we allowed correlations between group-level effects in the sprint model using an LKJ prior: $\text {LKJ}(1)$, which places uniform density over all correlation matrices.

For the endurance model, we used: $\beta _0 \sim \text {Normal}(0, 500)$ for the intercept, $(\beta _1, \beta _2, \beta _3) \sim \text {Normal}(0, 200)$ for the age coefficients (linear, quadratic, and cubic terms), and $\beta _{\text {test}} \sim \text {Normal}(0, 200)$ for test type effects (Interval Shuttle Run Test and Yo-Yo Intermittent Recovery Test 1). This intercept prior implies a 95% range of $\pm 1{,}000$ m around the mean-age value (observed: approximately 800–1,500 m across protocols), allowing the data to dominate inference. For group-level standard deviations, we specified $\sigma \sim \text {Exponential}(1)$ for study-level varying effects.

To facilitate model convergence in the vertical jump and endurance models, we set correlations between varying intercepts and slopes to zero when computing the covariance matrix. These priors were chosen to be weakly informative, providing mild regularization without substantially influencing posterior estimates while accounting for the measurement characteristics of each fitness indicator.

#### Missing Data

Studies varied in the range and completeness of their longitudinal observations. Missing observations arose from two principal sources: study-design-related gaps (not all studies assessed all fitness components at all time points) and participant attrition between measurement occasions—with 25% of included studies showing some concerns in attrition reporting as identified in the risk-of-bias assessment. The Bayesian multilevel growth curve model uses all available observed means—each study contributing to the likelihood only at the ages it measured—without imputation or interpolation. The polynomial growth curve and multilevel structure allow developmental trajectories to be estimated across the full age range by pooling information across studies, with posterior uncertainty naturally wider at ages where fewer studies contribute data.

#### Sensitivity Analysis

We conducted sensitivity analyses to assess the robustness of estimated developmental trajectories to the group-level effects structure and to the protocol subset used to define each outcome. For each fitness component, we compared models varying in both the covariates and the group-level effects specification. The criterion for a meaningful difference was non-overlap of the 90% posterior CrIs for the population-average APV across model variants; 68% CrIs are also reported to reflect a more sensitive threshold. Qualitative similarity of the trajectory shape was assessed by visual inspection of model-predicted curves across specifications.

For vertical jump, we compared a cubic polynomial trajectory with uncorrelated varying slopes (intercept, linear, quadratic, and cubic) by study and a test-type population-level effect (CMJ without vs. with arm swing); versus a cubic polynomial with uncorrelated varying slopes by sport × study interaction and a sport-level varying intercept, without a test-type population-level effect. The former model is the primary model reported in the main text, while the latter model tests whether absorbing between-sport variation at the varying effects level changes the estimated APV.

For sprint performance, we compared a quadratic trajectory with uncorrelated varying slope for age by study, fitted to 10-m data, with a linear trajectory with correlated varying slope by study plus a sport varying intercept, fitted to 10-m data—this linear model has no defined vertex and was assessed via approximate leave-one-out (LOO) and 10-fold cross-validation against the non-linear model. APV for the quadratic model was derived as described above.

For endurance performance, we compared a cubic polynomial with a test-type population-level effect (20-m Shuttle Test, Interval Shuttle Run Test, Yo-Yo IR1) and uncorrelated varying slopes by study—the primary model—against a cubic polynomial with a performance-type population-level effect (continuous vs. intermittent exercise) and uncorrelated varying slopes by study; and a model adding to the primary model uncorrelated varying slopes by sport. APV for these cubic models was derived as described above.

Across all outcomes, 90% posterior CrIs for APV overlapped across all model variants, and the qualitative shape of estimated developmental trajectories was consistent (see Supplementary Tables S2–S4 and Supplementary Figures S13–S15). These findings support the robustness of the developmental trajectories reported in the main text.

#### Model Diagnostics and Computation

We assessed model fit and validity using posterior predictive checks [31], comparing observed data to data simulated from the posterior predictive distribution. We examined trace plots, R-hat statistics (target < 1.01), and effective sample sizes to ensure chain convergence and adequate sampling. We used four Markov chains, each with 2000 iterations including 1000 warm-up iterations, for all models.

We fitted the Bayesian models using the R statistical language [32] and the brms package [33], which provides an interface to Stan [34]. We used the tidybayes package [35] to extract and manipulate posterior samples and the ggplot2 package [36] to visualize model predictions and developmental trajectories. All analysis code is available in the online repository.

## Results

### Study Selection and Characteristics

**Fig. 1:**
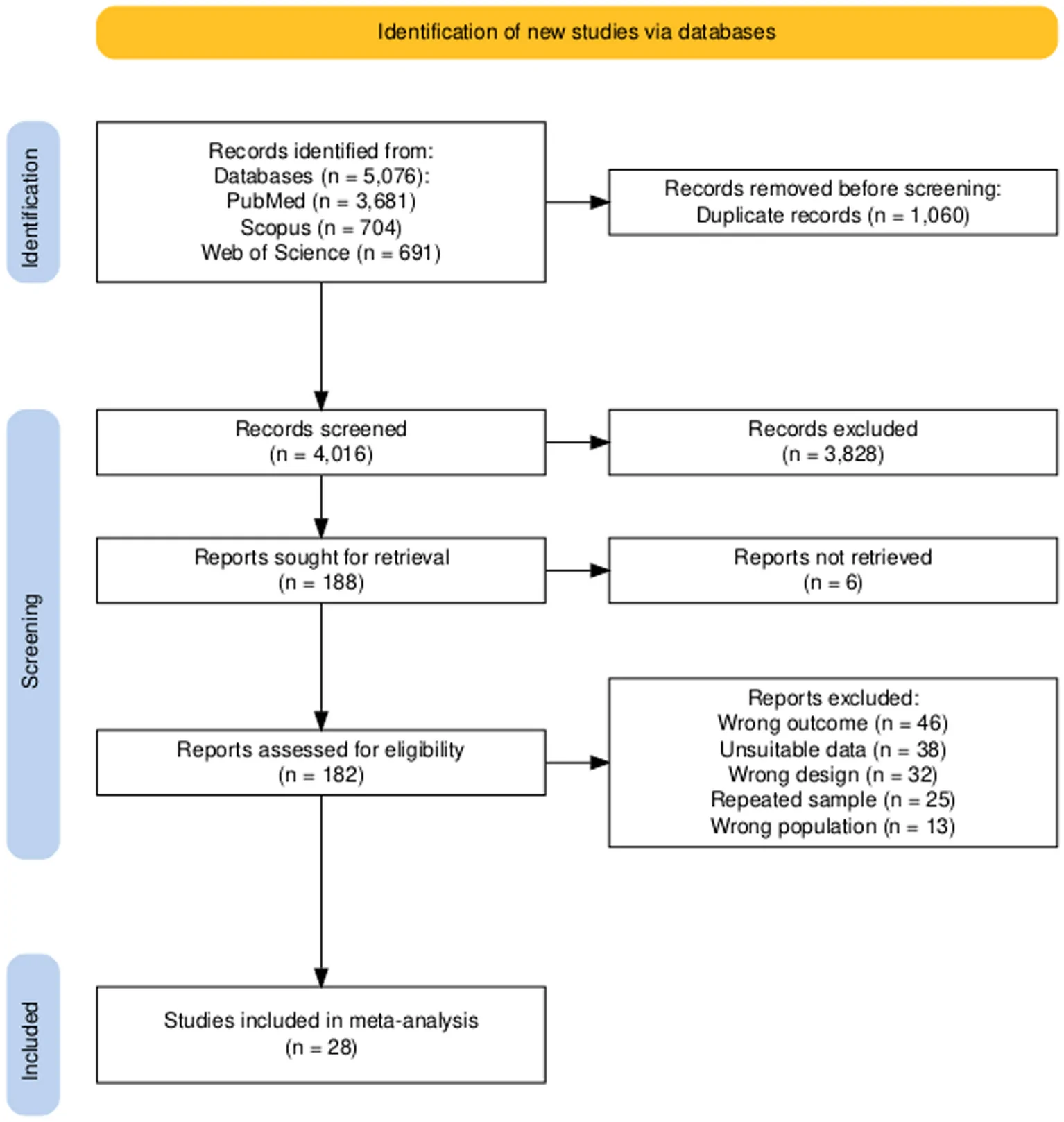
Flowchart including literature search and selection steps following the PRISMA statement

Following the systematic search and screening process (Fig. 1), 25 reports were excluded due to duplicate cohorts across publications, with the most comprehensive report retained; when publications from the same cohort reported distinct outcomes (e.g., one reporting sprint and another endurance), both were retained to maximize data coverage. An additional 38 studies were excluded due to insufficient data for analysis, including missing information on chronological age, assessment procedures, or outcome measures. Twenty-eight studies ultimately met the eligibility criteria for inclusion in the meta-analysis. Eight of these studies provided multiple independent samples, ranging from two to four groups within the same report, resulting in a total of 41 distinct samples for analysis. The distribution of fitness assessments across samples was as follows: 32 (22 studies) samples assessed vertical jump performance, 13 (8 studies) samples assessed 10-meter sprint performance, and 21 (15 studies) samples assessed endurance capacity using various protocols. Repeated samples—as labeled in Fig. 1—refer to publications identified at full-text review as reporting data from the same athlete cohort as another eligible publication. In such cases, the most comprehensive publication was retained. Where duplicate publications reported distinct fitness outcomes, both were retained to maximize outcome coverage. The 3,828 records excluded at the title/abstract screening stage were deemed clearly irrelevant to the eligibility criteria, consistent with standard PRISMA reporting practice at this stage.

The detailed characteristics of the 28 included studies are presented in the Supplementary Materials. The studies spanned multiple countries and nine sports overall, with football (soccer) being the most represented sport (*n* = 14 studies), followed by rugby (*n* = 5), basketball (*n* = 3), and single studies in handball, field hockey, ice hockey, tennis, volleyball, and freestyle wrestling (14 + 5 + 3 + 6 = 28 studies). Outcome-specific sport-stratified analyses (Figs. 3, 5 and 7) necessarily draw on subsets of these nine sports, since not every sport contributed data for every fitness component–vertical jump and sprint sport-specific models each included seven sports (rugby, football, handball, basketball, tennis, volleyball, and freestyle wrestling), while the endurance sport-specific model included six sports (football, basketball, handball, rugby, field hockey, and ice hockey), reflecting which sports had eligible data for each outcome. Study designs included pure longitudinal and mixed-longitudinal approaches, with durations ranging from 30 weeks to 11 years. Sample sizes ranged from 10 to 849 participants, with the number of repeated observations ranging from 3 to 12 measurement occasions. Most studies assessed vertical jump using the CMJ without arm swing, while sprint assessments focused primarily on 10-meter distances, and endurance tests included various protocols such as the Yo-Yo Intermittent Recovery Test Level 1, multistage fitness test, and interval shuttle run tests.

### Risk-of-Bias and Publication Bias Assessment

Risk-of-bias assessment revealed that included studies generally demonstrated good methodological quality (detailed assessment in Supplementary Materials). All studies adequately described study design, samples, and methods. Most domains showed low risk of bias, with some concerns noted primarily for analysis methods documentation (55% of studies) and attrition reporting (25% of studies). Overall, approximately 96% of studies had low risk of bias, with no studies rated as having high or critical risk, supporting the reliability of synthesized evidence.

Publication bias assessment through visual inspection of funnel plots and Bayesian Egger-type regression—using the model intercept as the parameter indicating small-study asymmetry, per convention—indicated evidence of asymmetry for 10-m sprint and endurance performance, but not for vertical jump (full model specification and distributional regression checks in Supplementary Materials). For vertical jump, the posterior mean intercept was 0.46 (68% CrI: -1.25; 2.20; k = 32 studies), with the interval spanning zero. For 10-m sprint, the posterior mean intercept was -6.10 (68% CrI: -9.80; -2.32; k = 13 studies), and for endurance performance 3.36 (68% CrI: 1.68; 5.08; k = 21 studies), with both intervals excluding zero. We report the presence of this asymmetry without attributing it to a specific direction of bias, as doing so would require additional assumptions about the underlying mechanism that cannot be verified from the available data. Pooled estimates for sprint and endurance performance should accordingly be interpreted with some caution; no such caution is indicated for vertical jump.

### Between-Study Heterogeneity

Between-study heterogeneity was quantified through the posterior distributions of the group-level effect standard deviations (σ), which decompose variability across levels of the multilevel structure. For vertical jump, the posterior mean σ for study-level intercepts was 5.15 cm (68% CrI: 4.51–5.78) and for linear slopes 0.83 cm/year (68% CrI: 0.65–1.01), indicating substantial between-study variability in absolute performance levels but considerably less heterogeneity in developmental rates. The quadratic and cubic slope standard deviations were negligible (0.09 and 0.01, respectively), suggesting that curve shape was broadly consistent across studies despite differences in absolute values. For 10-m sprint, analyses were restricted to a single test distance so no protocol moderator was required. The posterior mean σ for study-level intercepts was 0.12 s (68% CrI: 0.08–0.15) and for linear slopes 0.02 s/year (68% CrI: 0.01–0.03), indicating modest between-study variability in both absolute performance levels and developmental rates. The sport-level intercept standard deviation was 0.18 s (68% CrI: 0.07–0.27), exceeding the residual study-level variability and indicating that sport accounts for a meaningful proportion of between-study heterogeneity in absolute sprint performance. For endurance, the posterior mean (σ) for study-level intercepts was 106.34 m (68% CrI: 100.05–112.33) and for linear slopes 2.36 m/year (68% CrI: 0.23–3.66), reflecting substantial between-study variability in absolute performance levels consistent with the different test protocols employed. The cubic slope standard deviation was notably larger (13.56; 68% CrI: 11.39–15.70) relative to the fixed cubic effect, indicating meaningful between-study variability in the deceleration pattern in later adolescence. The sport-level intercept standard deviation was small (1.03 m; 68% CrI: 0.16–1.89), suggesting that sport differences in absolute endurance performance were largely accounted for by test protocol. Across all outcomes, between-study variability was greater in absolute performance levels than in developmental rates, supporting the use of sport- and protocol-stratified trajectories as the primary reporting approach.

### Vertical Jump Development

**Fig. 2:**
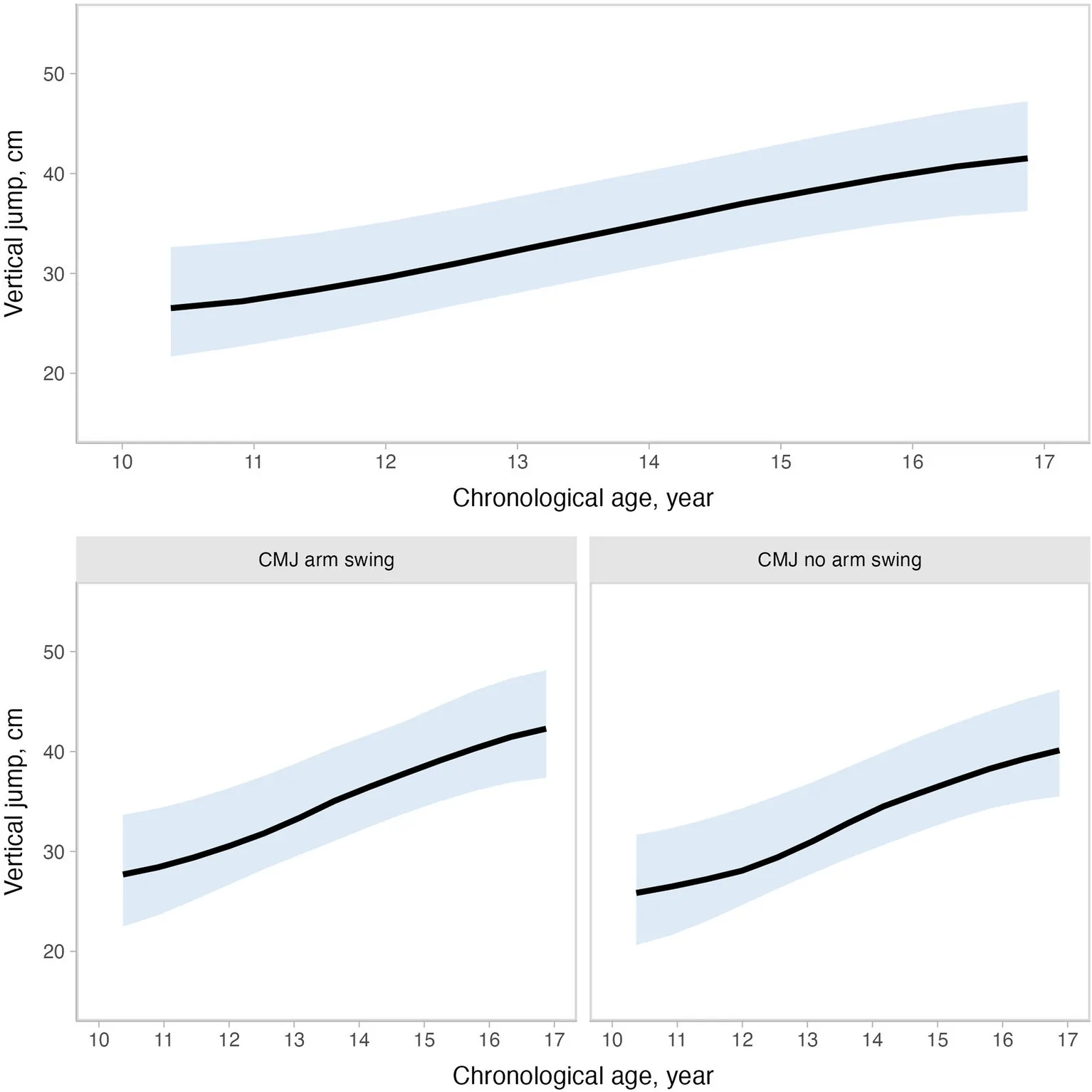
Meta-analysis predicted development of vertical jump performance by chronological age among young athletes (upper panel), and stratified by test protocol (lower panel). Error bands indicate the 68% credible interval

Figure 2 displays the population-level developmental trajectory for vertical jump performance across the age range of 10–17 years. The overall model revealed a consistent positive trajectory, with vertical jump height increasing from approximately 27 cm at age 10 to approximately 41 cm at age 17. The trajectory demonstrated a curvilinear pattern, with the rate of improvement appearing greatest during the early-to-mid adolescent years (ages 12–15) before showing some deceleration in later adolescence. Predicted trajectories with 68% CrI at each age are provided in Supplementary Table S5 for use as reference values in applied settings.

When stratified by test protocol (Fig. 2, lower panels), the CMJ with arm swing yielded higher absolute values throughout the age range compared to the CMJ without arm swing, as expected. The CMJ with arm swing trajectory ranged from approximately 28 cm at age 10 to 42 cm at age 17, while the CMJ without arm swing ranged from approximately 26 cm at age 10 to 40 cm at age 17. Despite these absolute differences, both protocols demonstrated similar developmental patterns across age, with parallel curvilinear trajectories. The uncertainty bands (credible intervals) were wider for the CMJ with arm swing protocol, reflecting the smaller number of studies using this assessment method. On average, APV for jump was 13.2 years (68% CrI, approximately ±1 posterior SD: 13.0–13.5).

**Fig. 3:**
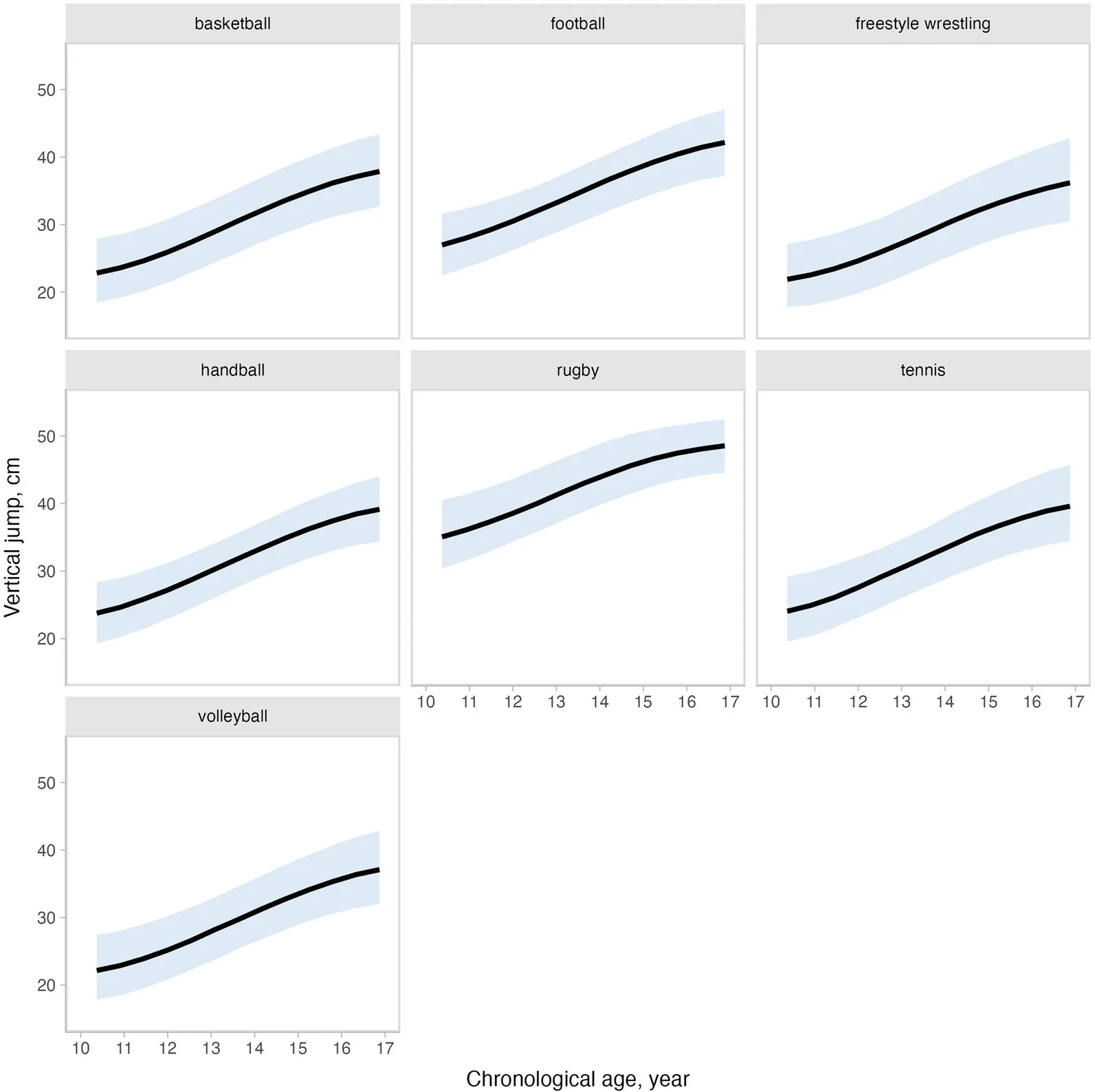
Meta-analysis predicted sport-specific development of vertical jump performance. Error bands indicate the 68% credible interval

Figure 3 presents sport-specific developmental trajectories for vertical jump performance. While all sports demonstrated positive developmental trends, notable between-sport differences were observed. Rugby showed the highest absolute vertical jump values across the age range, with performance increasing from approximately 35 cm at age 10 to nearly 49 cm at age 17. Football and handball showed similar trajectories, ranging from approximately 27 to 42 cm and 24 to 39 cm, respectively. Basketball, tennis, and volleyball demonstrated more modest absolute values but comparable developmental patterns. Freestyle wrestling showed lower initial values but substantial development across the observed age range. The uncertainty bands varied by sport, with narrower intervals for sports with larger sample sizes (e.g., football, rugby) and wider intervals for sports with fewer studies or smaller samples (e.g., freestyle wrestling, volleyball).

### Sprint Performance Development

**Fig. 4:**
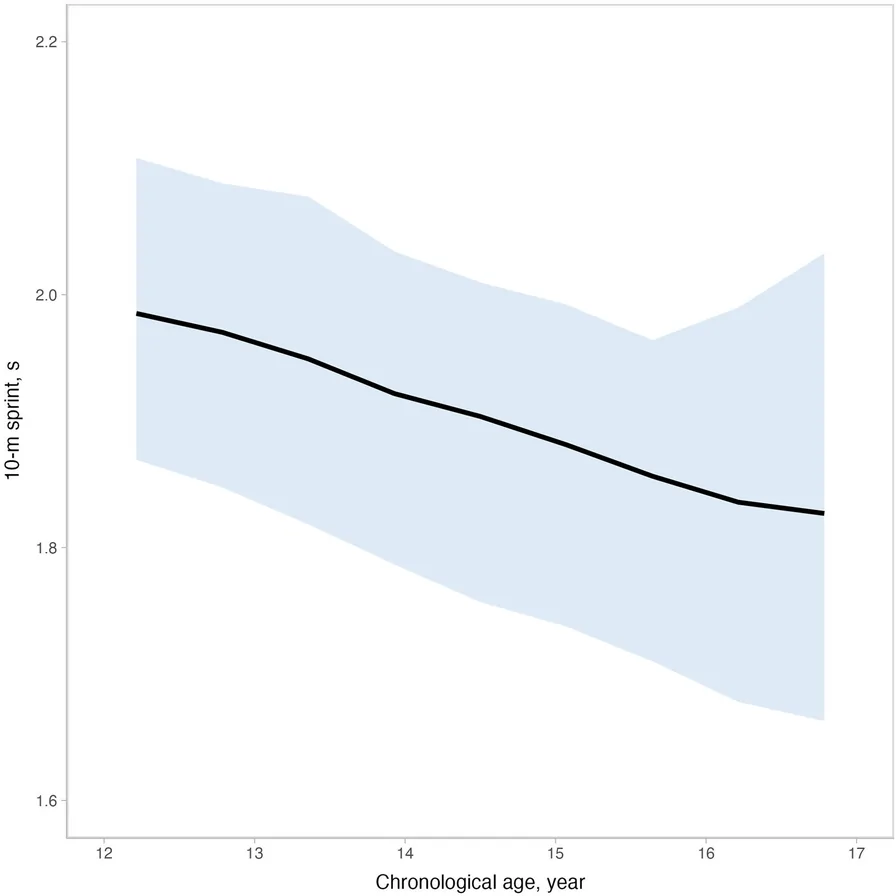
Meta-analysis predicted development of 10-m sprint performance by chronological age among young athletes. Error bands indicate the 68% credible interval

Figure 4 illustrates the developmental trajectory for 10-meter sprint performance from ages 12 to 17 years. Notably, sprint time decreased across age (indicating improved performance), with times decreasing from approximately 1.98 s at age 12 to approximately 1.83 s at age 17. The trajectory demonstrated a relatively linear pattern of improvement, with consistent decreases in sprint time throughout adolescence. The rate of improvement appeared relatively constant across the observed age range, without the pronounced curvilinear pattern observed for vertical jump or endurance performance. Predicted trajectories with 68% CrI at each age are provided in Supplementary Table S6 for use as reference values in applied settings.

**Fig. 5:**
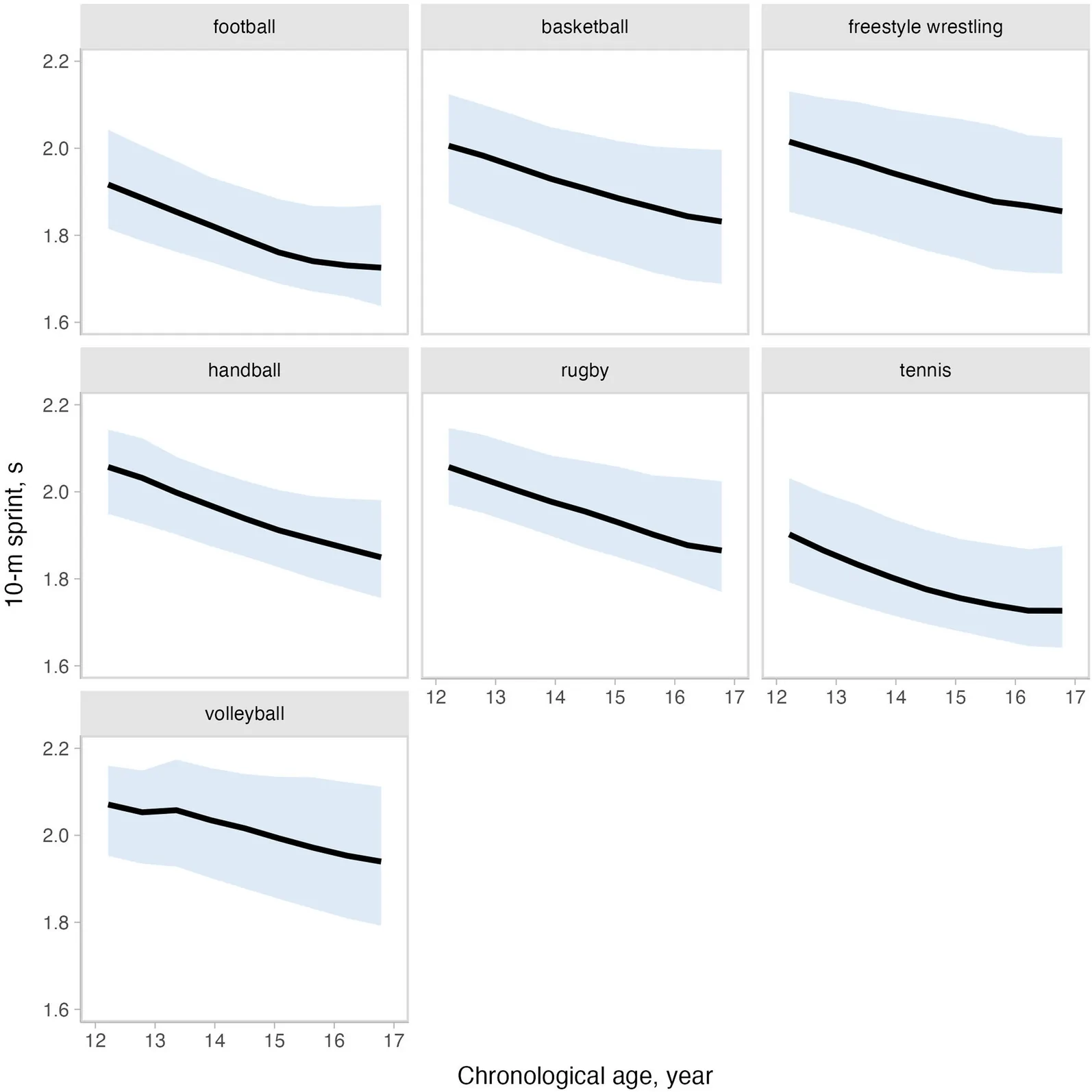
Meta-analysis predicted sport-specific development of 10-m sprint performance. Error bands indicate the 68% credible interval

Sport-specific trajectories (Fig. 5) revealed relatively similar patterns across different sports, with all showing linear improvements in sprint performance. Football, basketball, handball, rugby, and volleyball demonstrated comparable developmental trajectories with overlapping CrIs, suggesting minimal sport-specific differences in 10-m sprint development during adolescence. Tennis showed a similar pattern but with slightly wider uncertainty bands, reflecting the smaller sample size for this sport. Freestyle wrestling data were more limited, showing development primarily in the later adolescent years.

### Endurance Performance Development

**Fig. 6:**
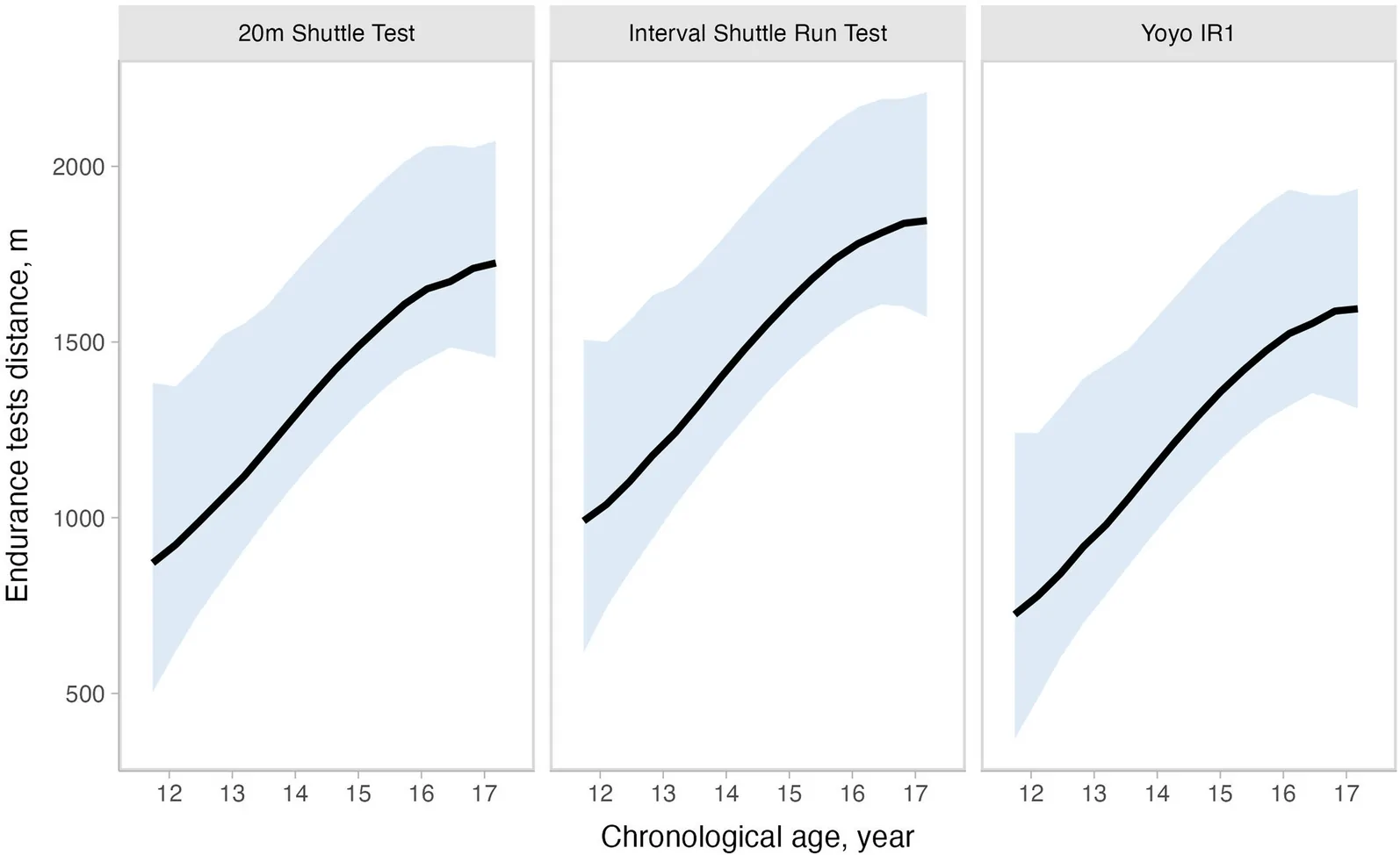
Meta-analysis predicted developmental trajectories for endurance performance stratified by test protocol. Error bands indicate the 68% credible interval

Figure 6 displays the developmental trajectories for endurance performance stratified by test protocol. All three endurance tests—the 20-m Shuttle Test, Interval Shuttle Run Test, and Yo-Yo Intermittent Recovery Test Level 1—demonstrated positive developmental trajectories across ages 12–17 years, with pronounced curvilinear patterns characterized by rapid improvements in early adolescence followed by continued but decelerating gains in later adolescence. On average, age at peak velocity for endurance development was estimated at 13.8 years (68% CrI: 13.6; 14.2). Predicted trajectories with 68% CrI at each age are provided in Supplementary Tables S7–S9 for use as reference values in applied settings.

The Interval Shuttle Run Test showed the highest absolute values, with performance increasing from approximately 1000 m at age 12 to approximately 1850 m at age 17. The Yo-Yo IR1 demonstrated a similar curvilinear pattern, ranging from approximately 700 m at age 12 to approximately 1600 m at age 17. The 20 m Shuttle Test trajectory ranged from approximately 900 to 1700 m across the same age range. All three protocols demonstrated the steepest rate of improvement between ages 12 and 15, with some evidence of plateauing or reduced rates of gain in later adolescence (ages 16–17). The uncertainty bands were widest for the 20-m Shuttle Test, likely reflecting greater between-study variability or smaller sample sizes compared to the other protocols.

**Fig. 7:**
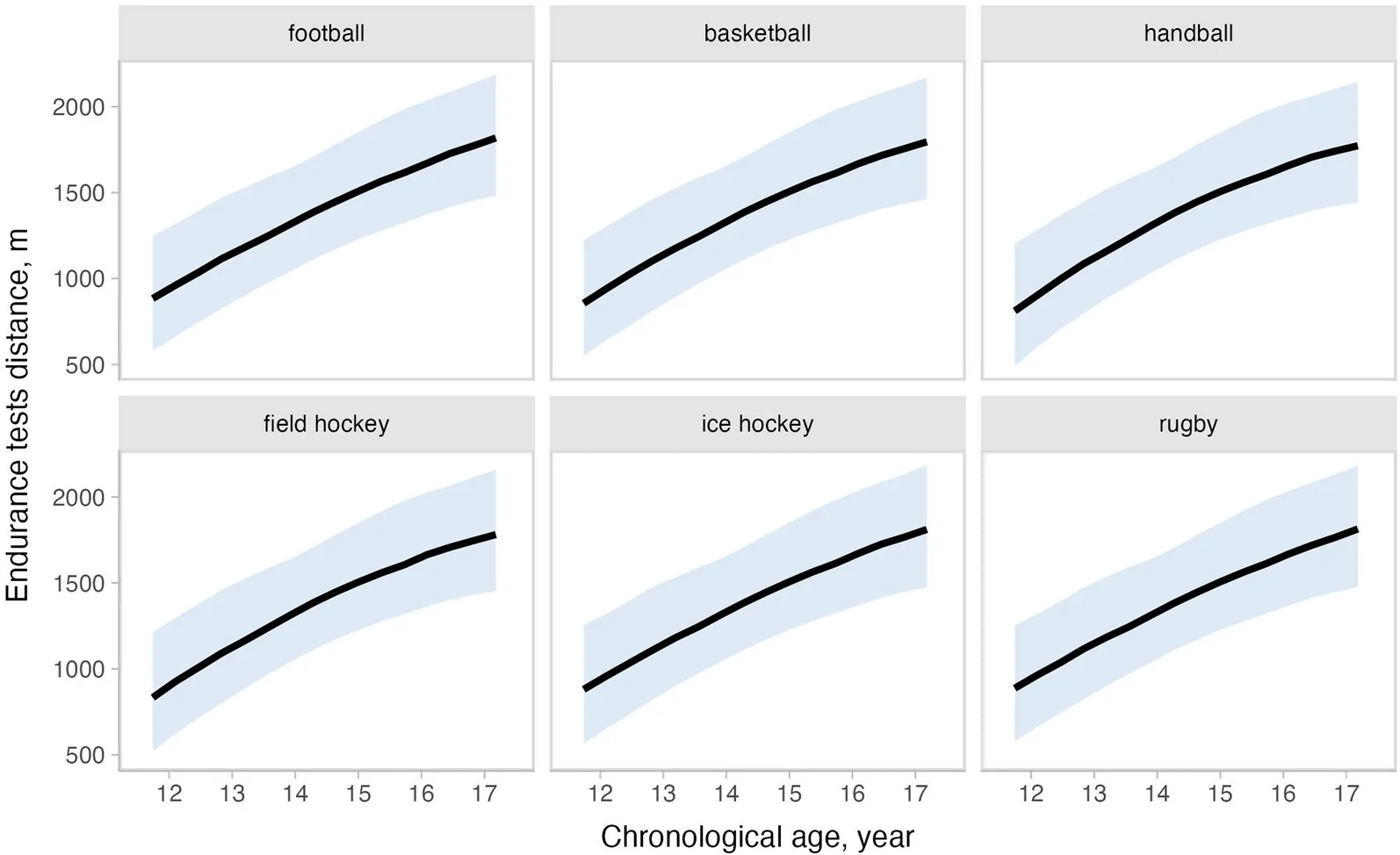
Meta-analysis predicted sport-specific developmental trajectories for endurance performance. Error bands indicate the 68% credible interval

Figure 7 presents sport-specific developmental trajectories for endurance performance. Football, basketball, and handball showed similar developmental patterns with substantial improvements across adolescence, all demonstrating a curvilinear trajectory with rapid early gains. Rugby and field hockey showed comparable patterns with slightly different absolute values. Ice hockey demonstrated a similar developmental trajectory but with wider CrIs, reflecting fewer studies in this sport. All sports showed pronounced curvilinear pattern of endurance development during adolescence, with peak rates of improvement occurring in early-to-mid adolescence.

## Discussion

This meta-analysis synthesized longitudinal data from multiple sports to estimate population-level developmental trajectories for vertical jump, sprint, and endurance performance in male youth athletes, providing age-referenced benchmarks for use in applied settings. Our findings reveal that these three fitness components follow distinct age-related developmental patterns throughout adolescence. Vertical jump and endurance performance exhibit pronounced curvilinear trajectories, with the steepest improvements occurring between ages 12 and 15, while sprint performance develops in a more linear fashion across the entire adolescent period. The timing of peak improvements for vertical jump (13.2 years) and endurance (13.8 years) overlaps the typical period of PHV attainment in adolescent male athletes. Summaries of large growth studies generally place PHV at approximately 14 years in boys [37, 38], while our previous longitudinal meta-analysis of young male athletes estimated a sport-specific PHV occurring about 1 year earlier (13.15 years) [17]. This temporal correspondence is a descriptive observation and should not be interpreted as evidence of a causal maturation effect. The relative contributions of biological maturation, chronological age, and cumulative training exposure cannot be disentangled in the present data: biological maturity was not assessed in the majority of included studies, and training exposure was not reported in extractable form. These absences mean that neither covariate adjustment nor alternative model specifications could address this confounding—it is a constraint of the available evidence base rather than of the analytical approach.

Progressive improvements in vertical jump performance during adolescence likely reflect multiple interacting factors. Our meta-analysis identified peak velocity for vertical jump at 13.2 years (68% CrI: 13.0; 13.5), coinciding with the typical period of rapid biological growth in adolescent male athletes [17]. During this period, increases in muscle strength, stature, body mass, and neuromuscular capacity are substantial [39–43]. Concurrently, accumulated training exposure, enhanced intersegmental coordination, technical refinement, and sport-specific practice also intensify and contribute importantly to performance gains [15, 16, 44–47]. The developmental curve offers a practical reference for interpreting progression and setting age-appropriate expectations, with the most substantial gains emerging during early-to-mid adolescence before plateauing toward age 17. Beyond this point, training-driven improvements continue but are likely small [48].

The CMJ provides a simple, reliable measure of neuromuscular function that captures lower-limb strength and power relevant to other explosive actions—particularly sprint performance [49–52]. In this meta-analysis, we included both CMJ protocols (with and without arm swing). As expected, CMJ with arm swing yielded slightly higher absolute values due to biomechanical advantages and intersegmental coordination [46, 53, 54]. However, separate developmental models showed very similar trajectory shapes and peak velocity ages, supporting pooling both protocols in the main analysis. When isolating lower-limb strength is the goal, CMJ without arm swing remains preferred.

Sport-specific curves revealed distinct absolute values despite broadly similar developmental patterns. Rugby exhibited the highest values, consistent with collision-based demands and resisted accelerations that progressively develop neuromuscular capacity [45, 55, 56]. Football, handball, and tennis showed intermediate trajectories, with football’s slightly higher values potentially reflecting cumulative training exposure and movement patterns specific to the sport [44, 57, 58]. Basketball and volleyball, despite frequent jump-dependent actions, showed lower absolute values likely related to technical specificity of sport-specific jumping mechanics rather than reduced power development [3, 16, 59]. Wrestling presented low initial values but steeper progression, likely reflecting substantial strength development during this age period [60]. Wider uncertainty intervals for volleyball and wrestling reflect limited longitudinal data availability, underscoring the need for additional monitoring. Overall, vertical jump development is shaped by biological growth, technical demands, training exposure, and sport-specific contexts.

Our meta-analysis showed that 10-m sprint performance improves in a steady and approximately linear trend throughout adolescence, with small but consistent yearly reductions in sprint time across the entire age range analyzed (12–17 years). This pattern indicates that, although the rate of improvement becomes slightly smaller after age 16, performance continues to progress without reaching a plateau. Sprint development did not show any abrupt acceleration or inflection point during the age period when rapid biological growth typically occurs. This trajectory is consistent with evidence showing that short-distance sprinting develops through gradual neuromuscular adaptations and progressive refinements in running technique [3, 44, 55, 57, 61]. These findings reinforce that early-acceleration performance follows a stable developmental pace throughout adolescence, further suggesting that young athletes likely reach their maximal sprint velocity only at later stages of development or the adult level.

Although the linear-quadratic and linear models showed comparable predictive performance in formal cross-validation—reflecting the limited power of the available sprint dataset to discriminate between closely related specifications—the visual attenuation of improvement at ages 16–17 (Fig. 4) is biologically plausible. Sprint performance is constrained by neuromuscular and biomechanical factors that approach maturity in late adolescence, and a gradual stabilization of improvement rates toward the end of the developmental period studied is consistent with evidence on neuromuscular development in youth athletes [62, 63]. The overall trajectory across ages 12–17 is therefore best described as predominantly linear with a tendency toward stabilization in late adolescence, rather than strictly linear throughout.

Sprint performance is closely related to neuromuscular power, with studies demonstrating strong associations between CMJ height, lower-body explosive strength, and short-distance acceleration in youth and adult athletes [4, 51, 52, 64]. This relationship indicates that the same neuromuscular qualities that drive improvements in jump performance, including maximal strength, rate of force development, and movement coordination, also support sprint development [4, 62]. However, because acceleration depends heavily on technical execution, motor control, and repeated practice under sport-specific constraints, these factors likely play a substantial role in the observed linear trajectory, with improvements accumulating progressively with training exposure rather than showing distinct acceleration during specific age periods [65].

The absence of meaningful sport-specific differentiation in short-distance sprint development warrants mechanistic consideration. We propose two non-exclusive explanations, which should be interpreted as hypotheses rather than confirmed findings. First, short-distance acceleration during adolescence may be primarily driven by general neuromuscular maturation—including progressive increases in muscle cross-sectional area, rate of force development, and motor unit synchronization [8, 41, 63]—that proceeds at broadly similar rates regardless of sport participation. If sprint performance during this developmental window is predominantly governed by these general neuromuscular qualities rather than by sport-specific technical adaptations [62, 64], comparable trajectories across sports would be expected. Second, the kinematic and kinetic determinants of sprint mechanics develop through accumulated running practice [62, 65], and running-based sports all provide substantial volumes of this practice during training and competition [44, 57, 61], constraining inter-sport divergence. These hypotheses cannot be confirmed in the absence of individual-level training content data and warrant direct investigation in future studies.

Short-distance sprinting is a fundamental determinant of performance across many youth sports, underpinning decisive actions in football, rugby, and tennis [56, 57, 66]. The predominantly linear developmental pattern observed here, with some tendency toward stabilization in late adolescence, underscores that acceleration capacity evolves progressively throughout adolescence, with incremental yet consistent annual gains serving as meaningful indicators of developmental progress. Coaches, strength and conditioning practitioners, and relevant stakeholders should therefore adjust their expectations, emphasizing year-to-year progression and training approaches that support the continuous accumulation of neuromuscular and technical adaptations rather than expecting distinct acceleration periods at specific ages.

Our meta-analysis revealed that endurance capacity follows a pronounced curvilinear age-related trajectory throughout adolescence, with the steepest improvements occurring between ages 12 and 15, before showing deceleration in later years. Age at peak velocity for endurance development was estimated at 13.8 years (68% CrI: 13.6; 14.2), closely corresponding with the typical age of PHV in male athletes and paralleling the trajectory observed for vertical jump performance.

This pattern is consistent with the substantial physiological changes in cardiorespiratory capacity that occur during adolescence [10, 67]. The years surrounding typical PHV timing coincide with marked increases in maximal oxygen uptake, enhanced cardiac stroke volume, greater capillary density, and improved oxidative enzyme capacity [68]. These physiological adaptations, combined with training exposure in structured sport environments, likely contribute to the rapid improvements in endurance performance observed during early-to-mid adolescence [8]. However, the extent to which these improvements reflect biological development versus training-related adaptations cannot be definitively determined from age-aligned data.

The developmental curves were remarkably consistent across the three test protocols (Yo-Yo IR1, 20 m Shuttle Test, Interval Shuttle Run Test), despite differences in absolute values reflecting test-specific characteristics. This suggests that the underlying age-related developmental pattern affects endurance capacity consistently regardless of whether the assessment emphasizes continuous or intermittent effort.

Sport-specific trajectories showed broadly similar patterns, though absolute values varied according to sport-specific demands and training emphases [69, 70]. These findings suggest that early-to-mid adolescence represents an important period for endurance development, when training adaptations may be particularly beneficial alongside ongoing physiological maturation.

### Limitations

Several limitations warrant consideration. First, our meta-analysis aligned performance data by chronological age rather than by biological maturity status, as almost all included studies—even at the primary-study level—did not assess maturity, at least using a valid maturity status indicator; the few datasets combining maturity assessment with longitudinal fitness monitoring [9, 30] are not publicly available for re-analysis. Individual variation in PHV timing (approximately ±1 year around the population mean; [17]) is therefore only partially captured within the 68% CrIs presented. Second, our analysis was restricted to male athletes. The database search was not limited by sex, but eligible female-athlete data remained insufficient to support separate analyses once the eligibility criteria—including the requirement for longitudinal, peer-reviewed, English-language reports—were applied. Findings are applicable exclusively to male athletes, as females may follow distinct maturation-related trajectories [8, 59]. Third, sport-specific trajectories for volleyball and freestyle wrestling showed wider uncertainty due to limited longitudinal data, warranting caution in applying these curves. Fourth, we could not account for training load, program structure, or cumulative training exposure, as this would require individual-level training data not reported in a standardized format across studies [15, 16], leaving it an uncontrolled source of variability and a structural constraint of aggregate-data meta-analysis rather than a specific shortcoming of the present study. Fifth, cross-protocol comparisons are valid for trajectory shape and timing but not for absolute performance values, as conversion between protocols was not performed. Sixth, publication bias assessment indicated an asymmetric relationship between study precision and effect size for 10-m sprint and endurance performance, but not for vertical jump; pooled trajectories for sprint and endurance should therefore be interpreted with some caution, with greater weight given to larger studies. Finally, attrition may bias observed trajectories. While voluntary dropout is unlikely to introduce systematic bias, coach-driven deselection of lower-performing athletes may progressively inflate performance estimates at older ages. Despite these limitations, this meta-analysis provides robust population-level reference curves from longitudinal data across multiple sports and countries.

### Practical Implications

The age-referenced trajectories and reference values provided in Supplementary Tables S5–S9 offer practical benchmarks for monitoring fitness progression and setting realistic expectations in youth sport contexts. Coaches can contextualize individual performance relative to population averages while recognizing substantial individual variation captured in the 68% CrI. The findings emphasize that physical fitness development reflects multiple interacting factors—biological growth, training exposure, technical refinement, and sport-specific demands—supporting long-term development approaches that prioritize progressive, individualized adaptation rather than early selection based on current performance.

Selected benchmarks illustrate the practical range: CMJ without arm swing is expected to increase from approximately 25.5 cm (68% CrI: 23.8–27.2) at age 10 to approximately 41.6 cm (68% CrI: 40.0–43.2) at age 17; 10-m sprint time decreases from approximately 2.07 s (68% CrI: 1.98–2.15) at age 12 to 1.83 s (68% CrI: 1.74–1.91) at age 17; and Yo-Yo IR1 performance increases from approximately 893 m (68% CrI: 813–968) at age 12 to approximately 1,610 m (68% CrI: 1,555–1,667) at age 17. The 68% CrI represents the range within which approximately two thirds of athletes of the same age and sport are expected to fall. Performance consistently below the lower bound may warrant additional monitoring attention, while performance above the upper bound may reflect early biological maturation or advanced training exposure. From a training periodization perspective, the curvilinear trajectories for vertical jump and endurance indicate that the fastest rates of improvement occur between ages 12–15, reflecting the concurrent influence of biological growth and training exposure, while the linear sprint trajectory indicates that acceleration capacity improves consistently throughout adolescence without a distinct period of accelerated gain.

## Conclusion

This meta-analysis provides robust, age-referenced developmental trajectories for vertical jump, sprint, and endurance performance in male youth athletes across multiple sports. The distinct patterns observed—curvilinear development for vertical jump and endurance versus linear progression for sprint—highlight that different physical capacities follow unique developmental trajectories during adolescence, with peak velocities coinciding with typical maturation timing.

## Supplementary Information

Supplementary Material 1

## Data Availability

All data presented in this review are available at https://osf.io/s6aeg/. All codes used to extract, model, and plot the data in this review are also available at https://osf.io/s6aeg/.

## Abbreviations

CMJ: Countermovement jump
PHV: Peak height velocity
APV: Age at peak velocity
CrI: Credible interval
IR1: Intermittent recovery level 1
PRISMA: Preferred Reporting Items for Systematic Reviews and Meta-Analyses
PROSPERO: International Prospective Register of Systematic Reviews
SD: Standard deviation
