# Supplementary material for: Development of Physical Fitness in Young Athletes: A Longitudinal Meta-analysis
Source: Sports Med Open. 2026 Sep 29;12:144. doi: 10.1186/s40798-026-01109-7 (PMC13624235; doi:10.1186/s40798-026-01109-7)
Supplement: Supplementary file 1 [file 40798_2026_1109_MOESM1_ESM.pdf]

Supplementary material for the manuscript: Development of physical fitness in young athletes: a longitudinal meta-analysis

Sports Medicine - Open

Ahlan B. Lima[1] Ricardo T. Quinaud[2] Gabriel F. Reis[3] Fábio C. Karasiak[1]
Eva Ageberg[4] Filipe M. Clemente[5,6,7] Carlos E. Gonçalves[8]
Humberto M. Carvalho[1,3,4]

1. School of Sports, Federal University of Santa Catarina, Florianópolis, Santa Catarina, Brazil; 2. Department of Physical Education, University of the Extreme South of Santa Catarina, Criciúma, Santa Catarina, Brazil; 3. Graduate Program in Psychology, Federal University of Santa Catarina, Florianópolis, Santa Catarina, Brazil; 4. Department of Health Sciences, Faculty of Medicine, Lund University, Lund, Sweden; 5. Polytechnic University of Coimbra, Coimbra, Portugal; 6. Sport Physical Activity and Health Research & Innovation Center, Applied Research Institute, Coimbra, Portugal; 7. Department of Biomechanics and Sport Engineering, Gdansk University of Physical Education and Sport, Poland; 8. Faculty of Sport Sciences and Physical Education, University of Coimbra, Coimbra, Portugal

This supplementary material provides comprehensive methodological details and additional analyses supporting the main manuscript on the development of physical fitness in young male athletes. The document is organized into four main sections:

- Study Characteristics (Supplementary Table S1) presents a detailed overview of the 28 longitudinal studies included in the meta-analysis, including sample sizes, study designs, measurement frequencies, and assessment protocols across different sports and countries.

- Risk of Bias Assessment evaluates the methodological quality of included studies using an adapted Cochrane risk of bias tool. We assessed eight domains relevant to longitudinal developmental research, with results presented both as aggregate summaries across domains (Figure S2) and detailed study-level judgments (Figure S3).

- Publication Bias Assessment examines potential small-study effects and publication bias through visual funnel plot inspection and Bayesian Egger-type regression for each fitness outcome (vertical jump, 10-m sprint, and endurance performance). An asymmetric relationship between study precision and effect size was evident for vertical jump and endurance performance but not for sprint; this section presents both individual-level residual analyses and study-level posterior estimates to comprehensively evaluate the presence of systematic bias in the literature.

- Posterior Predictive Checks evaluate the adequacy of our Bayesian multilevel models by comparing observed data distributions with posterior predictive distributions. These diagnostic plots confirm that our models appropriately capture the central tendency, variability, and distributional characteristics of developmental trajectories across all three fitness outcomes.

- Reference Values (Supplementary Tables S5–S9) present predicted trajectories with 68% credible intervals (CrIs) at each half-year age increment from 10 to 17 years for vertical jump, sprint, and endurance performance, for use as age-referenced benchmarks in applied settings.

- Sensitivity Analysis (Supplementary Tables S2–S4) evaluates the robustness of estimated developmental trajectories by comparing alternative model specifications for each fitness outcome. Exact protocol subsets, the criterion for meaningful differences (overlap of 68% and 90% posterior CrIs for the age at peak development velocity), and a quantitative summary of population-average estimates across model variants are provided.

Together, these supplementary materials provide transparency regarding study inclusion, methodological quality assessment, bias evaluation, model validation, sensitivity of conclusions to modelling choices, and practical reference benchmarks, supporting the robustness and interpretability of the developmental trajectories reported in the main manuscript.

Study Characteristics

Supplementary Table S1 summarizes the characteristics of the 28 longitudinal studies synthesized in this meta-analysis. The included studies span multiple countries across Europe, South America, Asia, and Oceania, with the majority conducted in European nations. Football was the most represented sport (n = 14 studies), followed by rugby (n = 5), basketball (n = 3), and single studies in volleyball, handball, tennis, field hockey, ice hockey, and freestyle wrestling (nine sports represented overall; 14 + 5 + 3 + 6 = 28 studies). Sample sizes ranged from 10 to 849 participants, with observation periods varying from 30 weeks to 11 years. Most studies employed mixed-longitudinal designs (n = 16), combining cross-sectional age cohorts with longitudinal follow-up, while 12 used purely longitudinal designs following single cohorts over time. The number of measurement occasions ranged from 3 to 12 time points per study. Vertical jump was assessed in 22 studies, endurance performance in 15 studies, and 10-meter sprint in 8 studies. Assessment protocols included countermovement jump with and without arm swing, various endurance tests (Yo-Yo Intermittent Recovery Test Level 1, Multistage Fitness Test, Interval Shuttle Run Test), and 10-meter sprint timing. This diverse evidence base provides broad representation across sports, developmental periods, and geographical contexts, enhancing the generalizability of the synthesized developmental trajectories.

Figure S1 displays the raw study-level mean trajectories for each fitness component across the observed age range. Each line represents one independent sample, with colors distinguishing studies. Study-level means are shown without uncertainty bounds; apparent non-monotonic patterns in individual studies reflect sampling variability around the underlying developmental trend rather than true reversals in performance. Model-predicted trajectories, which account for this variability through the multilevel structure, are presented in the main text figures. The full observed age range per study is shown; model predictions are restricted to ages 10–17 for vertical jump and endurance and 12–17 for sprint, reflecting the range with adequate data coverage across studies.

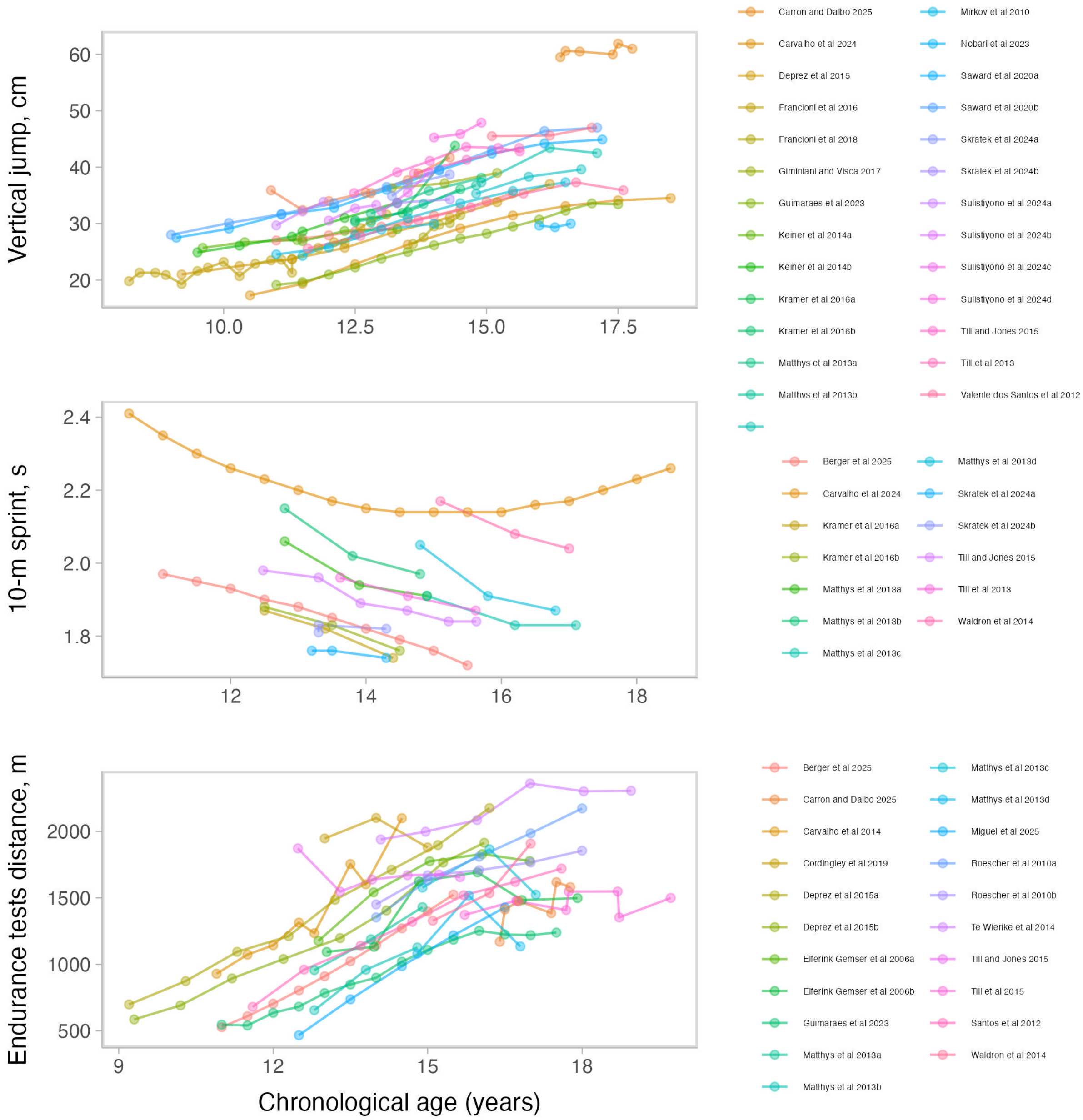

Figure S1: Study-level mean trajectories show observed group means at each measurement occasion without uncertainty bounds; apparent non-monotonic patterns in individual studies reflect sampling variability around the underlying developmental trend rather than true reversals in performance. Model-predicted trajectories, which account for this variability through the multilevel structure, are presented in the main text figures.

Table S1: Characteristics of the longitudinal studies included in the meta-analysis.

Study	Country	Sport	Sample size*	Design	Duration	Obs.	Fitness indicator	Assessment
Berger et al. (2015)	Brazil	Football	76	Mixed-long.	1.5 years	6	VJ, Sprint, End.	CMJ no arm, 10m, Yo-Yo IR1
Bidaurrazaga-Letona et al. (2015)	Spain	Football	37	Mixed-long.	4 years	8	VJ	CMJ no arm
Carron and Dalbo (2025)	Australia	Rugby	11	Longitudinal	1 year	6	VJ, End.	CMJ no arm, MSFT
Carvalho et al. (2014)	Spain	Football	29	Mixed-long.	4 years	8	Endurance	Yo-Yo IR1
Carvalho et al. (2024)	Brazil	Volleyball	40	Mixed-long.	1 year	4	VJ, Sprint	CMJ no arm, 10m
Cordingley et al. (2019)	Canada	Ice hockey	62	Longitudinal	2 years	3	Endurance	MSFT
Deprez et al. (2015)	Belgium	Football	89	Longitudinal	2 years	3	VJ, End.	CMJ no arm, Yo-Yo IR1
Elferink-Gemser et al. (2006)	Netherlands	Field hockey	29	Mixed-long.	2 years	3	Endurance	ISRT grass
Francioni et al. (2016)	Italy	Football	27	Mixed-long.	30 weeks	4	VJ	CMJ no arm
Francioni et al. (2018)	Italy	Football	33	Longitudinal	10 months	6	VJ	CMJ no arm
Giminiani and Visca (2017)	Italy	Football	19	Longitudinal	2 years	3	VJ	CMJ no arm
Guimarães et al. (2023)	Portugal	Basketball	293	Mixed-long.	2.5 years	6	VJ, End.	CMJ no arm, Yo-Yo IR1
Keiner et al. (2014)	Germany	Football	19	Longitudinal	2 years	3	VJ	CMJ no arm
Kramer et al. (2016)	Netherlands	Tennis	45	Mixed-long.	7 years	8	VJ, Sprint	CMJ arm, 10m
Matthys et al. (2013)	Belgium	Handball	39	Longitudinal	2 years	3	VJ, Sprint, End.	CMJ arm, 10m, Yo-Yo IR1
Miguel et al. (2025)	Brazil	Basketball	44	Mixed-long.	2.5 years	8	VJ, End.	CMJ no arm, Yo-Yo IR1
Mirkov et al. (2010)	Serbia	Football	26	Longitudinal	3 years	4	VJ	CMJ no arm
Nobari et al. (2023)	Turkey	Wrestling	10	Longitudinal	32 weeks	3	VJ	CMJ no arm
Roescher et al. (2010)	Netherlands	Football	28	Mixed-long.	5 years	5	Endurance	ISRT
Santos et al. (2012)	Portugal	Football	83	Mixed-long.	4 years	5	VJ, End.	CMJ no arm, MSFT
Saward et al. (2020)	UK	Football	849	Mixed-long.	11 years	12	VJ	CMJ arm
Skratek et al. (2024)	Germany	Football	48	Longitudinal	1 year	3	VJ, Sprint	CMJ no arm, 10m
Sulistiyono et al. (2024)	Indonesia	Football	17	Mixed-long.	1 year	3	VJ	CMJ arm
Te Wierike et al. (2014)	Netherlands	Basketball	31	Mixed-long.	2 years	6	Endurance	ISRT
Till and Jones (2015)	UK	Rugby	67	Mixed-long.	5 years	6	VJ, Sprint, End.	CMJ no arm, 10m, MSFT
Till et al. (2013)	UK	Rugby	81	Longitudinal	3 years	4	VJ, Sprint	CMJ no arm, 10m
Till et al. (2015)	UK	Rugby	44	Mixed-long.	5 years	6	Endurance	Yo-Yo IR1
Waldron et al. (2014)	UK	Rugby	21	Longitudinal	2 years	3	VJ, Sprint, End.	CMJ no arm, 10m, MSFT

* Sample size refers to the maximum number of participants observed across time points.
Abbreviations: VJ = Vertical jump; End. = Endurance; Mixed-long. = Mixed-longitudinal; Obs. = Number of observations; CMJ no arm = Countermovement jump without arm swing; CMJ arm = Countermovement jump with arm swing; 10m = 10-meter sprint; MSFT = Multistage fitness test; ISRT = Interval shuttle run test; UK = United Kingdom.

Risk of Bias Assessment

We adapted the Cochrane risk of bias tool for randomized trials to assess bias in our longitudinal observational studies, focusing on eight domains relevant to longitudinal developmental research. Two reviewers independently assessed risk of bias for each study, with disagreements resolved through discussion. Figure S2 presents a summary of risk of bias judgments across all domains, and Figure S3 provides detailed study-level assessments.

Assessment Domains

The adapted tool evaluated the following domains:

D1: Study Design Adequacy - Adequate description of cohort, inclusion/exclusion criteria, and sport context

D2: Sample Description - Completeness of participant characteristic reporting

D3: Methodological Description - Detail and clarity of measurement protocols

D4: Attrition and Dropout - Reporting of dropout rates, loss to follow-up, and handling of missing data

D5: Outcome Measurement Reliability - Use of standardized and validated measurement protocols

D6: Results Reporting Adequacy - Completeness of reporting for data extraction purposes

D7: Consistency Across Multiple Reports - Consistency of design, sample, and results across publications from the same cohort

D8: Analysis Methods Appropriateness - Appropriateness of statistical approaches for longitudinal data

Results by Domain

For Study Design (D1) all 28 included studies (100%) were rated as having low risk of bias for study design adequacy (Figure S2). Each study provided clear descriptions of their cohort composition, explicit inclusion and exclusion criteria, and sufficient detail about the sport context, competitive level, and training environment. Study-level judgments are presented in Figure S3.

For Sample Description (D2) all 28 studies (100%) received low risk ratings for sample description (Figure S2). Studies comprehensively reported participant characteristics including age at each measurement occasion, sample sizes, and relevant demographic information.

For Methodological Description (D3) all 28 studies (100%) demonstrated low risk of bias for methodological description (Figure S2). Studies provided detailed protocols for fitness assessments, including specific test procedures, equipment used, and measurement conditions.

For Attrition and Dropout (D4) more variability was observed in risk assessment (Figure S2). Twenty-one studies (75%) were considered as low risk, adequately reporting participant retention, explicitly documenting dropout rates, and describing handling of missing data or loss to follow-up. Seven studies (25%) showed some concerns due to incomplete reporting of attrition rates, unclear documentation of which participants were measured at each time point, or insufficient description of how missing data were handled in analyses. No studies were rated as high risk in this domain.

Outcome Measurement Reliability (D5). The reliability of outcome measures was generally strong (Figure S2). Approximately 24 studies (85%) were classified as low risk. The studies used standardized, validated fitness assessment protocols with clear descriptions of measurement procedures, trained assessors, and quality control procedures. Four studies (15%) showed some concerns related to insufficient details about measurement standardization, limited information about assessor training or reliability, or unclear conditions under which tests were administered. No studies were rated as high risk.

Results Reporting Adequacy (D6). Most studies provided sufficient data for meta-analysis inclusion (Figure S2). A total of 22 studies (80%) provided comprehensive descriptive statistics (means, standard deviations or standard errors) for all measurement occasions, enabling straightforward data extraction. Six studies (20%) showed some concerns due to incomplete reporting of descriptive statistics at certain time points, presentation of data primarily in graphical form requiring digitization, or missing information about sample sizes at specific measurement occasions. No studies were rated as high risk.

Consistency Across Multiple Reports (D7). This domain was not applicable to most studies, as the majority represented independent cohorts with single publications (Figures S2-S3). Fourteen

studies (50%) were standalone publications from unique cohorts. One study (4%) with multiple publications from the same cohort showed consistent reporting of design, sample characteristics, and overlapping results. Thirteen studies (46%) showed minor inconsistencies in reporting across multiple publications from the same cohort or had unclear relationships with other publications, making it difficult to assess consistency fully.

Analysis Methods Appropriateness (D8). This domain showed the most variability across studies (Figure S2). Thirteen studies (45%) used appropriate longitudinal statistical methods, adequately accounted for repeated measures, adjusted for relevant confounders (particularly biological maturation), and provided sufficient statistical detail. Fifteen studies (55%) showed some concerns related to limited statistical detail, use of cross-sectional approaches for longitudinal data, absence of adjustment for maturation or other key confounders, or unclear handling of the nested structure of repeated measures within individuals. No studies were rated as high risk, though several showed methodological limitations that warranted caution in interpretation.

Detailed study-level risk of bias judgments for all eight domains are presented in Figure S3.

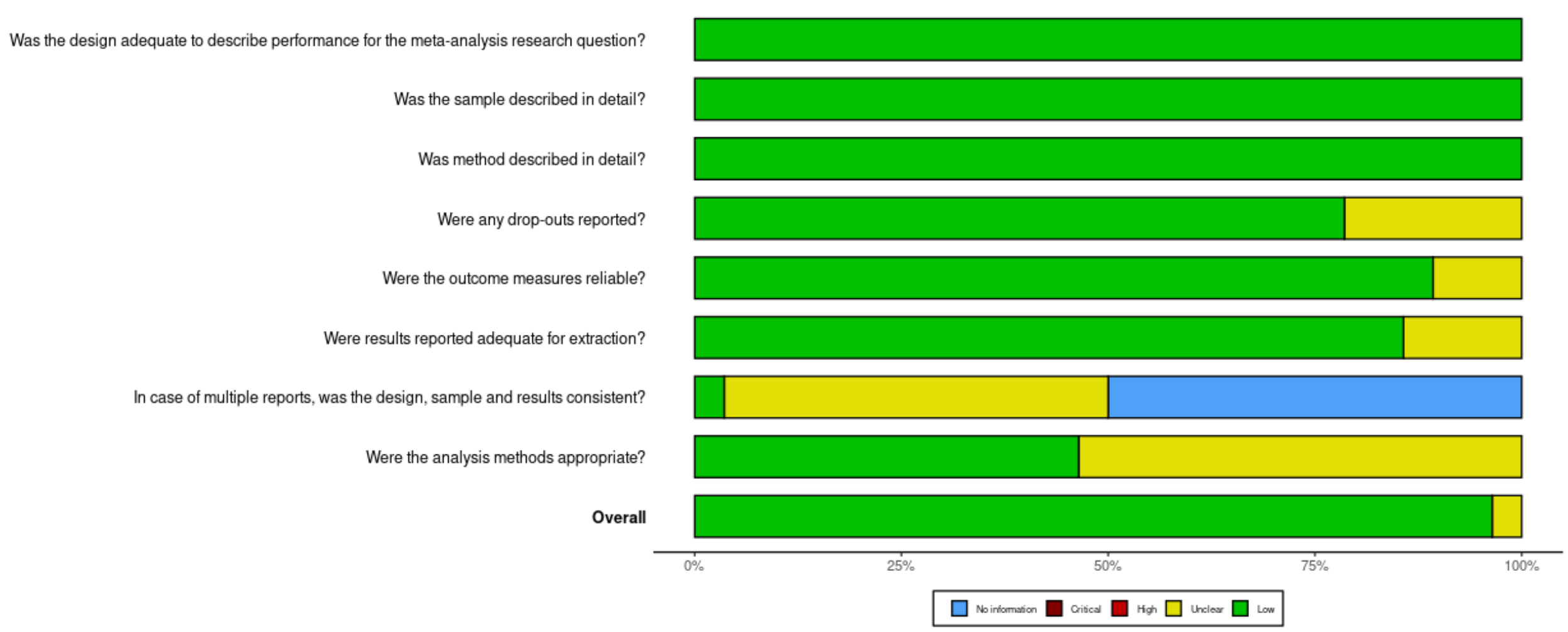

Figure S2: Summary of risk of bias assessments across eight domains for all included studies. Green indicates low risk of bias, yellow indicates some concerns or unclear risk, blue indicates not applicable (primarily for Domain 7: consistency across multiple reports), and red indicates high risk of bias.

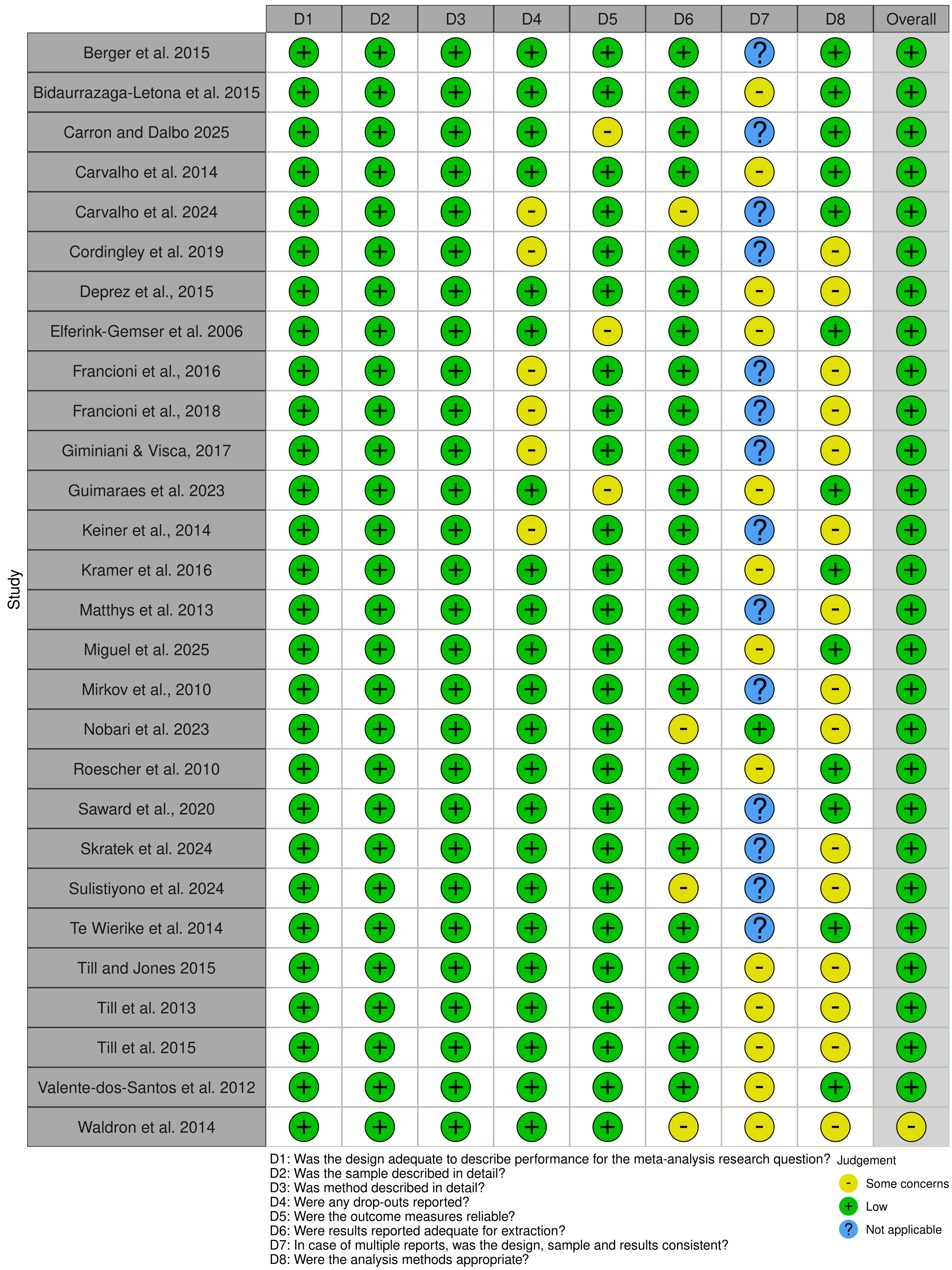

Figure S3: Study-level risk of bias assessments across all eight domains. Each row represents an individual study, with symbols indicating risk of bias judgment for each domain: + (green) = low risk, - (yellow) = some concerns, ? (blue) = not applicable/unclear, and (red) = high risk. D1–D8 correspond to the eight assessment domains described in the text.

Overall Risk of Bias

The overall risk of bias was determined by considering patterns across all eight domains (Figures S2-S3). A total of 27 studies (96%) demonstrated low risk of bias overall, with either all domains rated as low risk or minor concerns in only one or two domains that were unlikely to substantially affect the validity of developmental trajectory estimates. One study (4%) showed some concerns overall due to multiple domains with unclear or concerning ratings, though not rising to the level of high risk. No studies (0%) were rated as having high or critical overall risk of bias.

The risk of bias assessment indicates that the body of evidence synthesized in this meta-analysis is of generally high quality. The included studies demonstrated particular strengths in study design documentation, sample description, and methodological transparency. The most common limitations related to incomplete reporting of attrition and dropout patterns, variability in the sophistication of longitudinal analytical approaches, and challenges in assessing consistency across multiple reports from the same cohorts.

The absence of studies with high overall risk of bias, combined with the preponderance of studies with low risk across critical domains (study design, outcome measurement, results reporting), provides confidence that the developmental trajectories estimated in this meta-analysis are not substantially biased by methodological limitations in the primary studies. However, the concerns noted for analytical methods in 55% of studies highlight the continuing need for more rigorous application of appropriate longitudinal statistical methods in youth sport science research.

Publication Bias Assessment

We examined potential publication bias through visual inspection of residual plots and a Bayesian Egger-type regression, regressing each study's standardized effect (effect divided by its standard error) on the inverse standard error, with a multilevel structure accounting for repeated observations within study. Because included studies contributed effects across a developmental age range rather than a single estimate, we additionally included an age-centered × inverse-standard-error interaction term to separate genuine developmental signal from precision-related asymmetry. The model intercept is the parameter conventionally interpreted as indicating small-study effects or

publication bias; the slope on inverse standard error is not interpreted, consistent with standard practice in the meta-analysis literature. As a complementary check targeting a related but distinct question, we additionally examined a distributional regression of individual-level model residuals against inverse sample size, which assesses whether residual variance itself differs systematically by study size.

Small-Study Effects: Vertical Jump

The posterior mean intercept for vertical jump was 0.46 (68% CrI: $-$1.25; 2.20) across k=32 studies, with the interval spanning zero and indicating no evidence of small-study asymmetry. Funnel plots (Supplementary Figures S4-S5) are presented for visual inspection.

Small-Study Effects: 10-m Sprint

The posterior mean intercept for 10-m sprint was $-$6.10 (68% CrI: $-$9.80; $-$2.32) across k=13 studies, with the interval excluding zero and indicating evidence of asymmetry between study precision and effect size. The complementary distributional regression model (applied to individual-level residuals, n=3,150) did not show a comparable pattern, with a posterior mean $\sigma_{\text{inverse n}}$ of 6.23 (68% CrI: 5.22; 7.24) consistent with expected sampling variability rather than a systematic relationship between study size and residual variance. We report this divergence between the two approaches transparently: the Egger-type regression and the distributional regression test related but distinct forms of bias, the former targeting whether study precision predicts effect magnitude, the latter targeting whether study size predicts residual variability, and a finding from one need not be corroborated by the other. We therefore do not draw a single unified conclusion about publication bias for sprint performance, and present both results for transparency.

Small-Study Effects: Endurance Performance

The posterior mean intercept for endurance performance was 3.36 (68% CrI: 1.68; 5.08) across k=21 studies, with the interval excluding zero and indicating evidence of asymmetry between study precision and effect size. The complementary distributional regression model (applied to individual-level residuals, n=6,250) corroborated this pattern, with a posterior mean $\beta_{\text{inverse n}}$ of 405.54 (68% CrI: 333.30; 477.78), indicating that residual variance also differed systematically

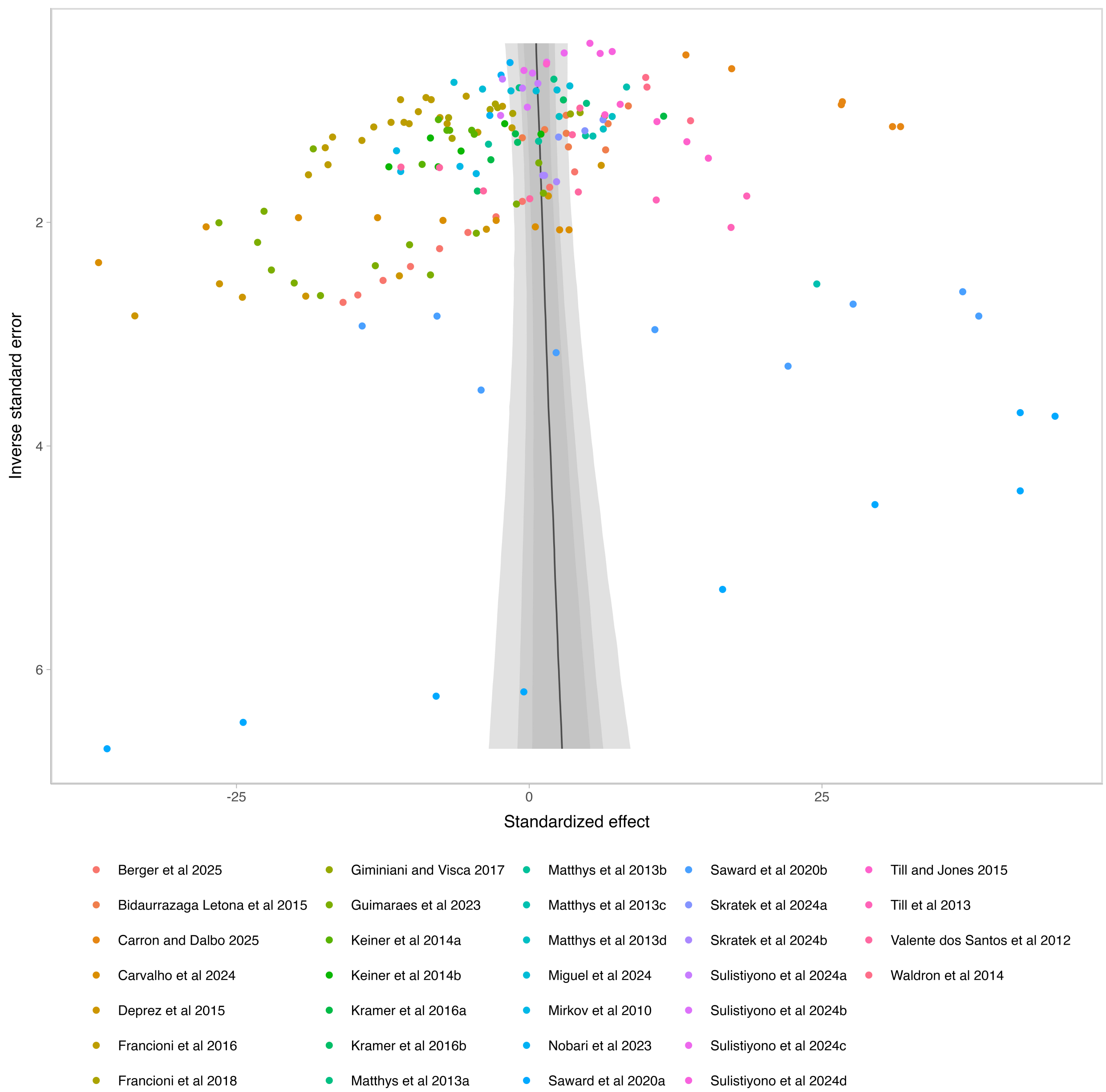

Figure S4: Residual plot for vertical jump performance, displaying standardized errors against model residuals for each study sample.

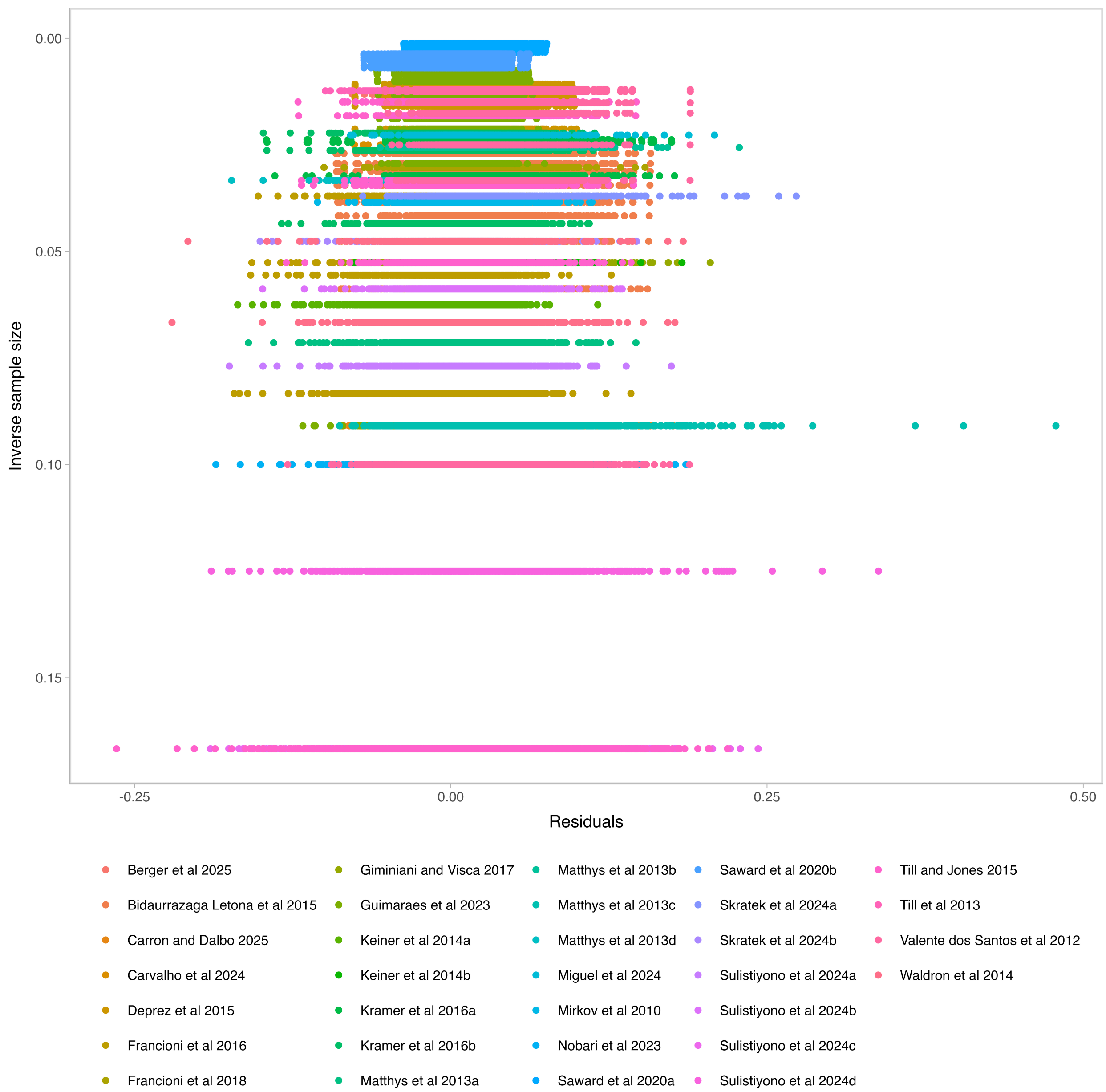

Figure S5: Residual plot for vertical jump performance, showing residuals plotted against inverse sample size for each study sample.

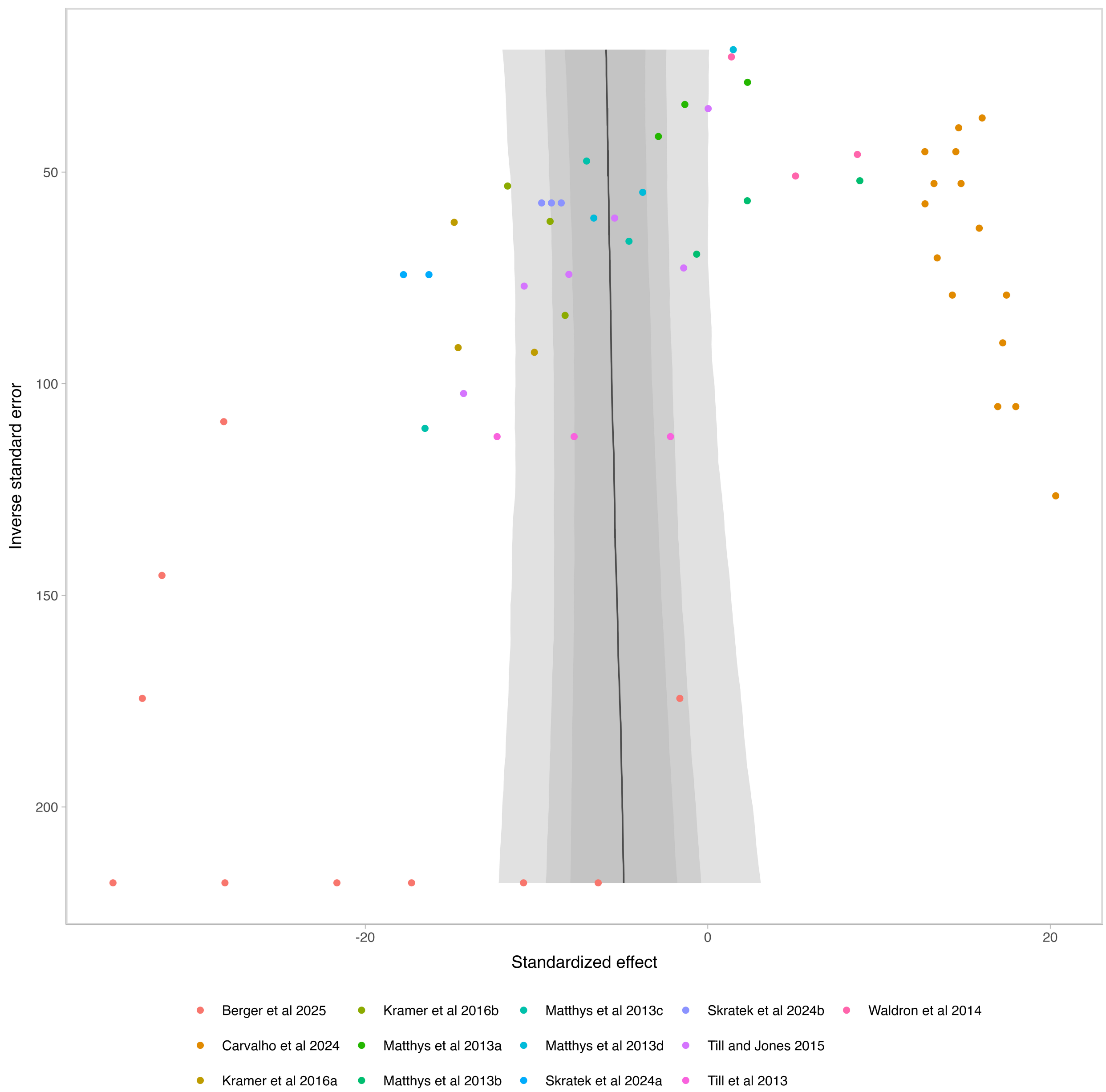

Figure S6: Residual plot for sprint performance, displaying standardized errors against model residuals for each study sample.

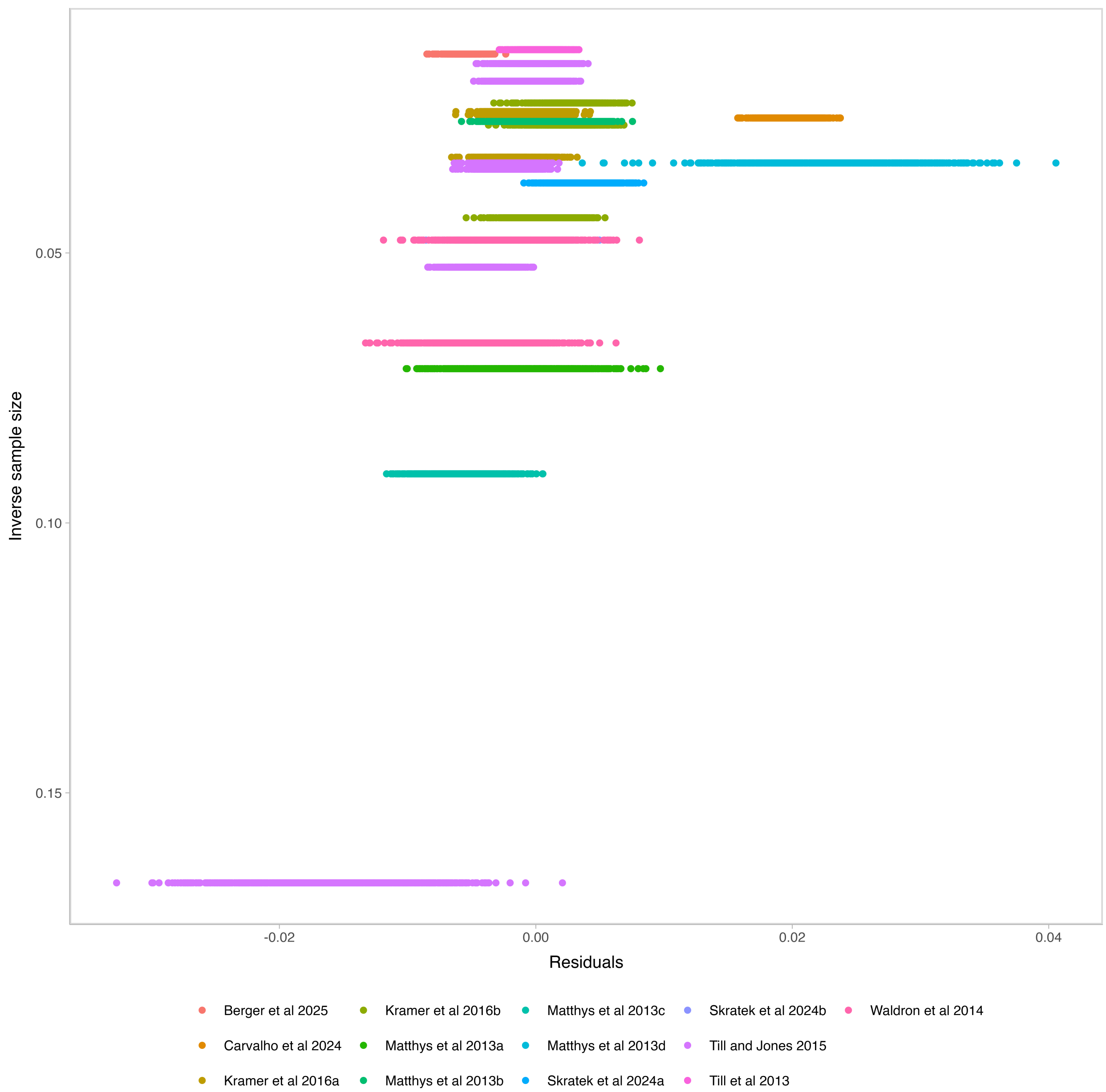

Figure S7: Residual plot for sprint performance, showing residuals plotted against inverse sample size for each study sample.

by study size. Both approaches therefore converge on evidence of small-study asymmetry for endurance performance. Pooled estimates for this outcome should accordingly be interpreted with some caution, with greater weight given to larger, more methodologically rigorous studies.

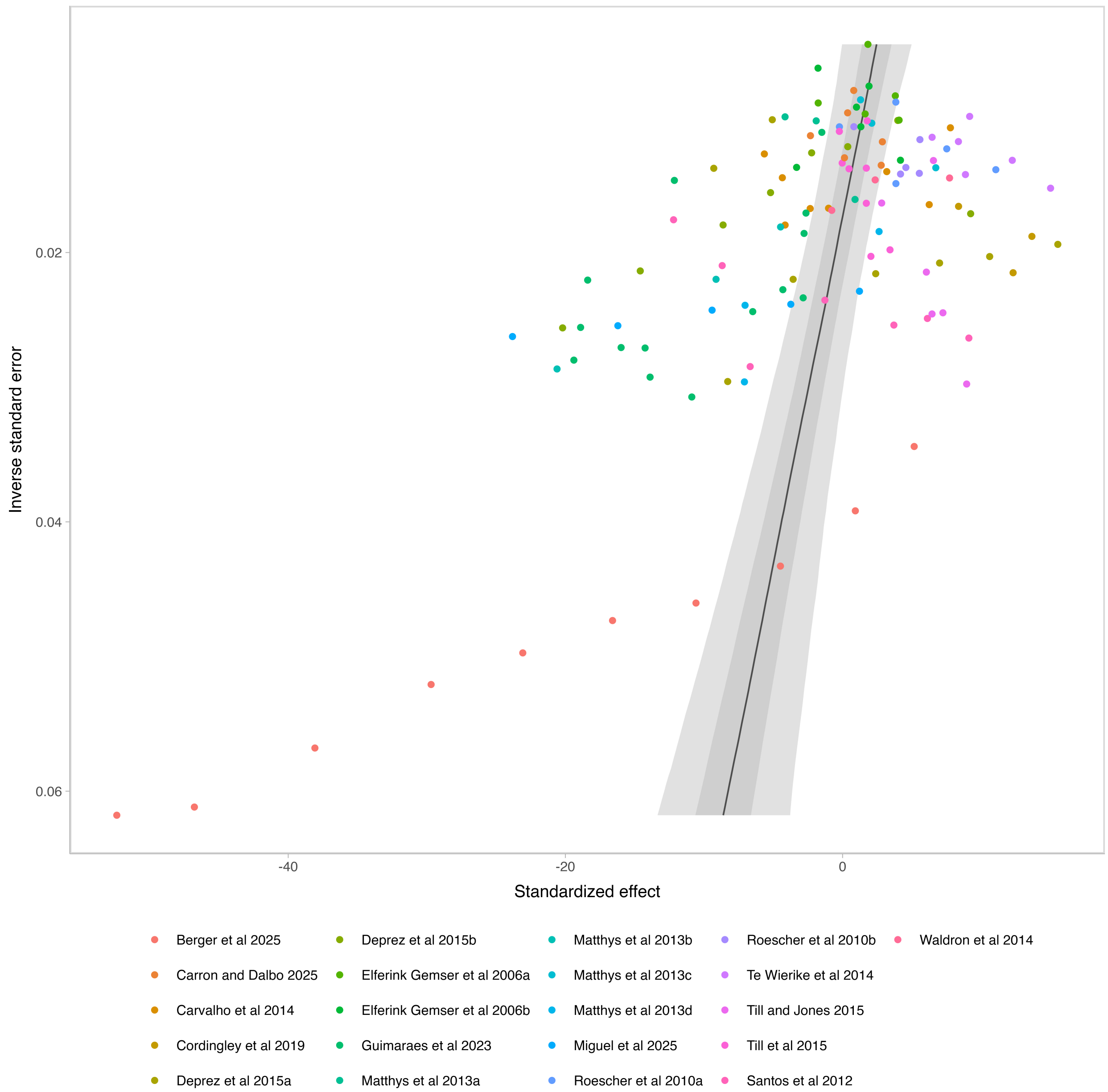

Figure S8: Residual plot for endurance performance, displaying standardized errors against model residuals for each study sample.

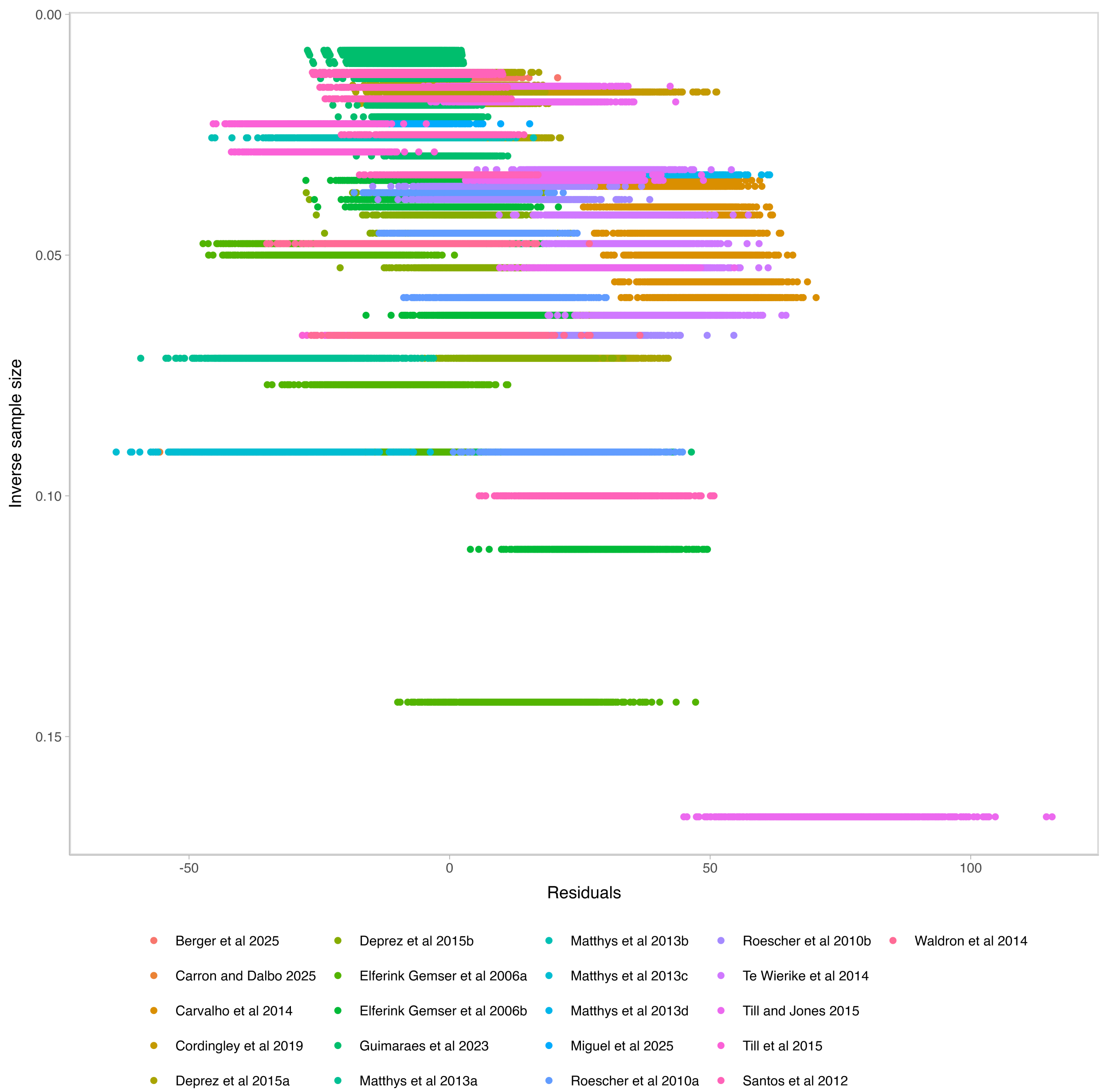

Figure S9: Residual plot for endurance performance, showing residuals plotted against inverse sample size for each study sample.

Posterior Predictive Checks

Posterior predictive checks assess model adequacy by comparing the distribution of observed data against distributions generated from the fitted model. Good model fit is indicated when the observed data distribution falls within the range of plausible replications from the posterior predictive distribution. All three models demonstrate satisfactory fit, with observed data patterns well-replicated by the posterior predictive distributions, supporting the validity of the estimated developmental trajectories.

The figure displays the distribution of observed data (y, dark blue line) overlaid with 100 posterior predictive replications (`y_rep`, light blue lines). The close alignment between the observed data distribution and the posterior predictive distributions indicates good model fit. The bimodal pattern in the residual distribution reflects the clustering of data points around zero (well-fitted observations) with some spread in the tails, consistent with expected between-study and within-individual variability in longitudinal trajectories.

The observed data distribution (dark blue) shows strong correspondence with posterior predictive replications (light blue). The model successfully captures the primary peak centered near zero, indicating adequate representation of the central tendency and spread of residuals. The secondary smaller peak around 0.3 in the posterior predictive distributions reflects the model's uncertainty in capturing some heterogeneity in sprint development patterns across studies, though the overall fit remains acceptable.

The posterior predictive distributions (light blue) closely match the observed data distribution (dark blue), demonstrating strong model fit for endurance trajectories. The approximately symmetric, bell-shaped distribution centered near zero indicates that the Bayesian multilevel model appropriately captures the central tendency and variability in endurance development across studies and individuals. The close overlap across the full range of residuals suggests the model adequately accounts for both within-individual correlation and between-study heterogeneity in endurance performance trajectories.

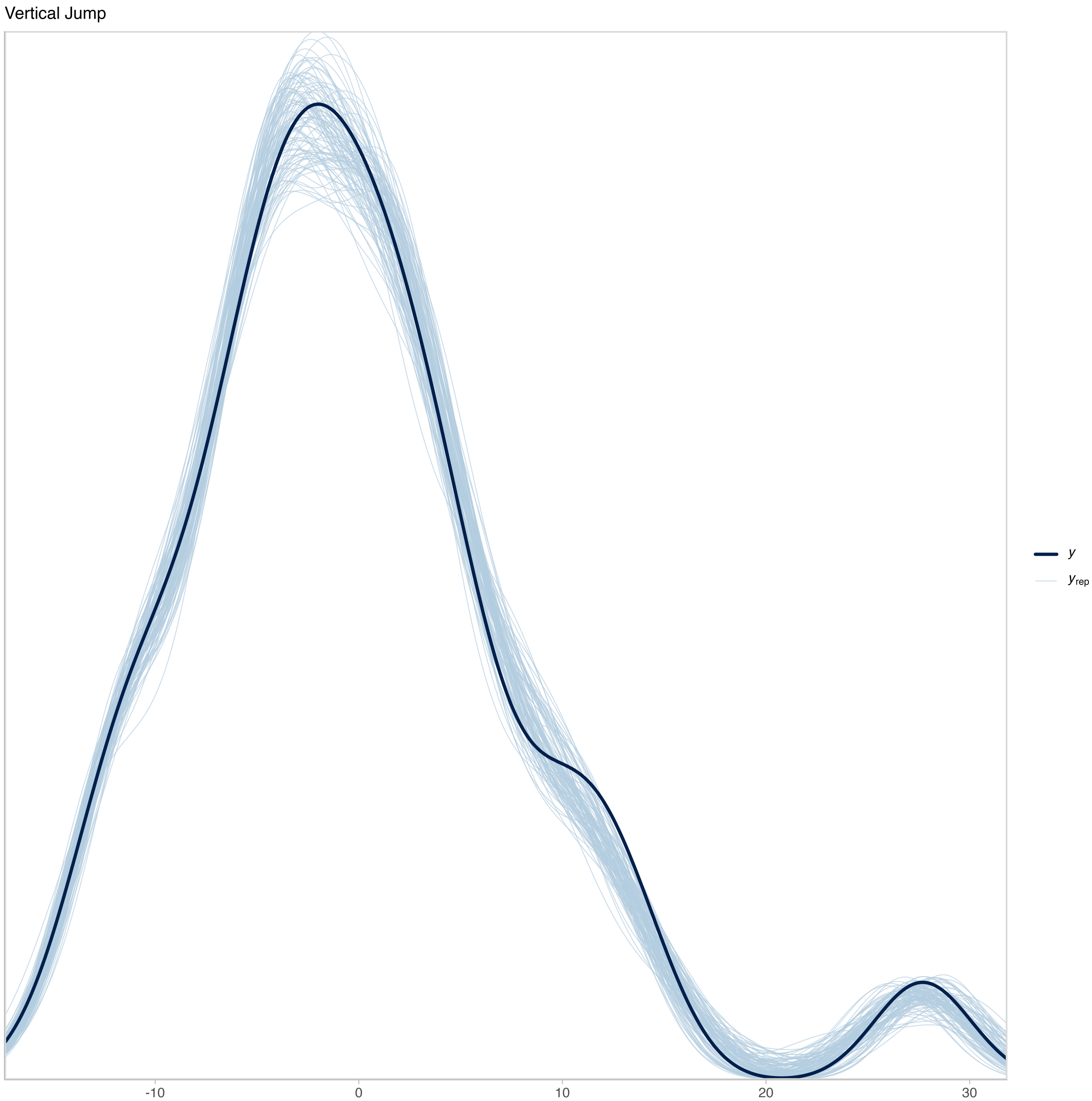

Figure S10: Posterior predictive check for vertical jump performance model.

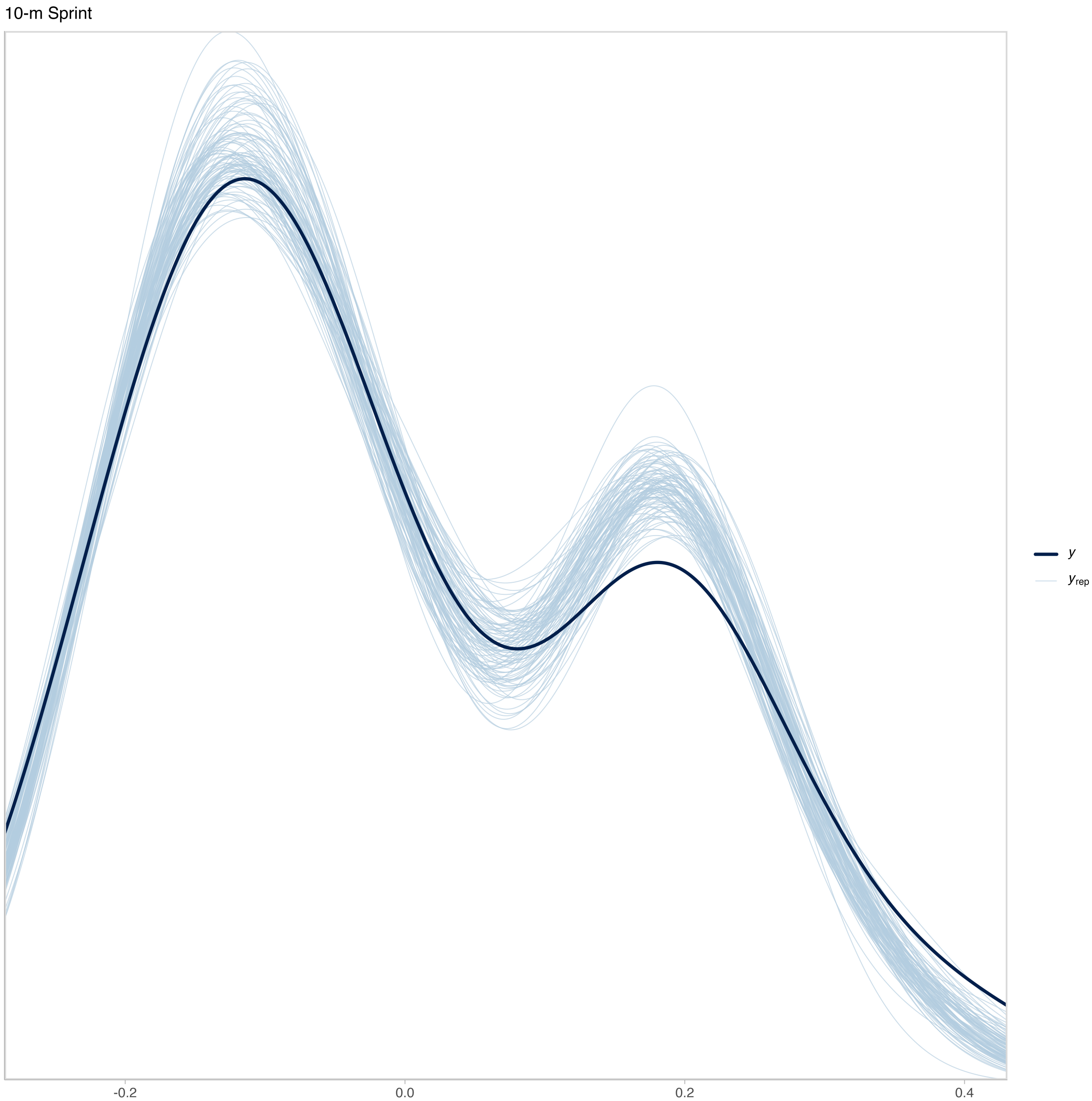

Figure S11: Posterior predictive check for 10-m sprint performance model.

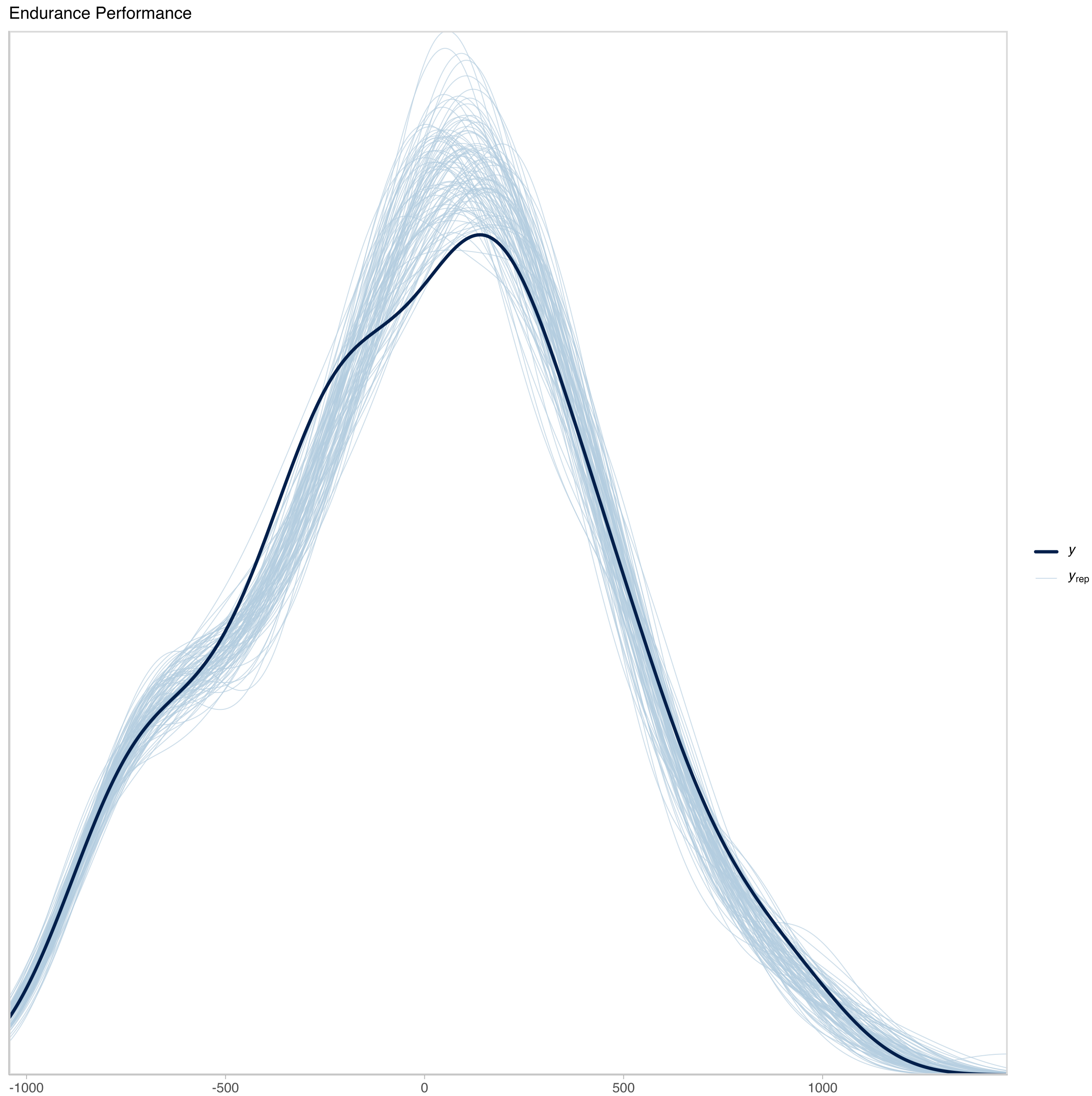

Figure S12: Posterior predictive check for endurance performance model.

Sensitivity Analysis

We assessed the robustness of estimated developmental trajectories by fitting alternative Bayesian multilevel model specifications for each fitness outcome, varying the group-level effects structure and the covariate set for protocol subsets. For outcomes modelled with a cubic polynomial (vertical jump, endurance), the population-average age at peak development velocity (APV) was derived analytically as the vertex of the cubic's first derivative: $\text{APV} = -(b_{\text{age}^2} \times 2) \,/\, (b_{\text{age}^3} \times 6) + \bar{x}_{\text{age}}$. For sprint (quadratic model), the age at minimum sprint time (i.e., peak sprint performance) was the vertex of the quadratic: $\text{APV} = -b_{\text{age}} \,/\, (2 \times b_{\text{age}^2}) + \bar{x}_{\text{age}}$. The criterion for a meaningful difference was non-overlap of the 90% posterior CrIs for APV across model variants; 68% CrIs are also reported to reflect a more sensitive threshold. Qualitative similarity of the trajectory shape was assessed visually from the model-predicted curves (main text figures).

Vertical Jump

Two model specifications were compared:

- M2 (primary model): cubic polynomial trajectory; uncorrelated varying slopes (intercept, linear, quadratic, cubic) by study; test-type population-level effect (CMJ without arm swing vs. CMJ with arm swing).
- M3 (sensitivity): cubic polynomial trajectory; uncorrelated varying slopes by sport × study interaction; sport-level varying intercept. This tests whether absorbing between-sport variance in level changes the estimated population-average APV.

Supplementary Table S2 presents the posterior mean APV and 68% and 90% CrIs for each specification. The 90% CrIs overlapped between M2 and M3, indicating that the estimated age at peak jump development velocity was robust to the inclusion of sport-level varying effects in the multilevel structure.

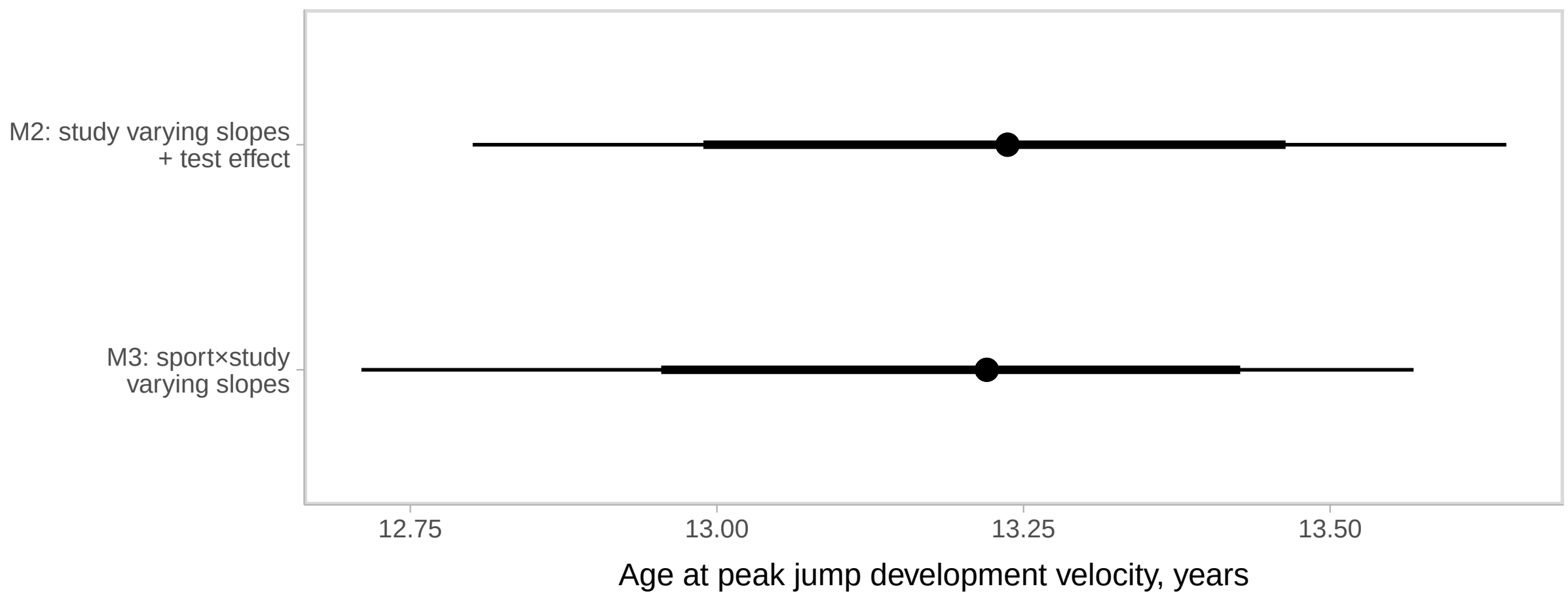

Figure S13: Sensitivity analysis for vertical jump: population-average age at peak development velocity (APV) from two model specifications. Points indicate posterior means; thick and thin bars show 68% and 90% posterior credible intervals, respectively.

Table S2: Sensitivity analysis for vertical jump: population-average age at peak development velocity (APV) from two model specifications with 68% and 90% posterior credible intervals.

Model	Mean APV (years)	68% CrI	90% CrI
M2: study varying slopes + test effect	13.23	[12.99, 13.46]	[12.8, 13.64]
M3: sport×study varying slopes	13.19	[12.95, 13.43]	[12.71, 13.57]

Sprint Performance

Five distance-specific model specifications were compared:

- f2 (primary, 10 m): quadratic trajectory ($\text{age} + \text{age}^2$); uncorrelated varying slope for age by study.
- f2.1 (sensitivity, 10 m): linear trajectory (age only); correlated varying slope by study; sport varying intercept. Because this model is linear, it has no vertex and APV is not defined; sensitivity was assessed via approximate LOO cross-validation and 10-fold cross-validation comparing f2 and f2.1.

- f3 (primary, 20 m): quadratic trajectory; correlated varying slope for age by study.
- f4 (primary, 30 m): quadratic trajectory; correlated varying slopes (age, age^2) by study.
- f5 (sensitivity, 30 m): as f4 plus sport varying intercept. Tests whether absorbing between-sport variation changes the APV for 30-m sprint.

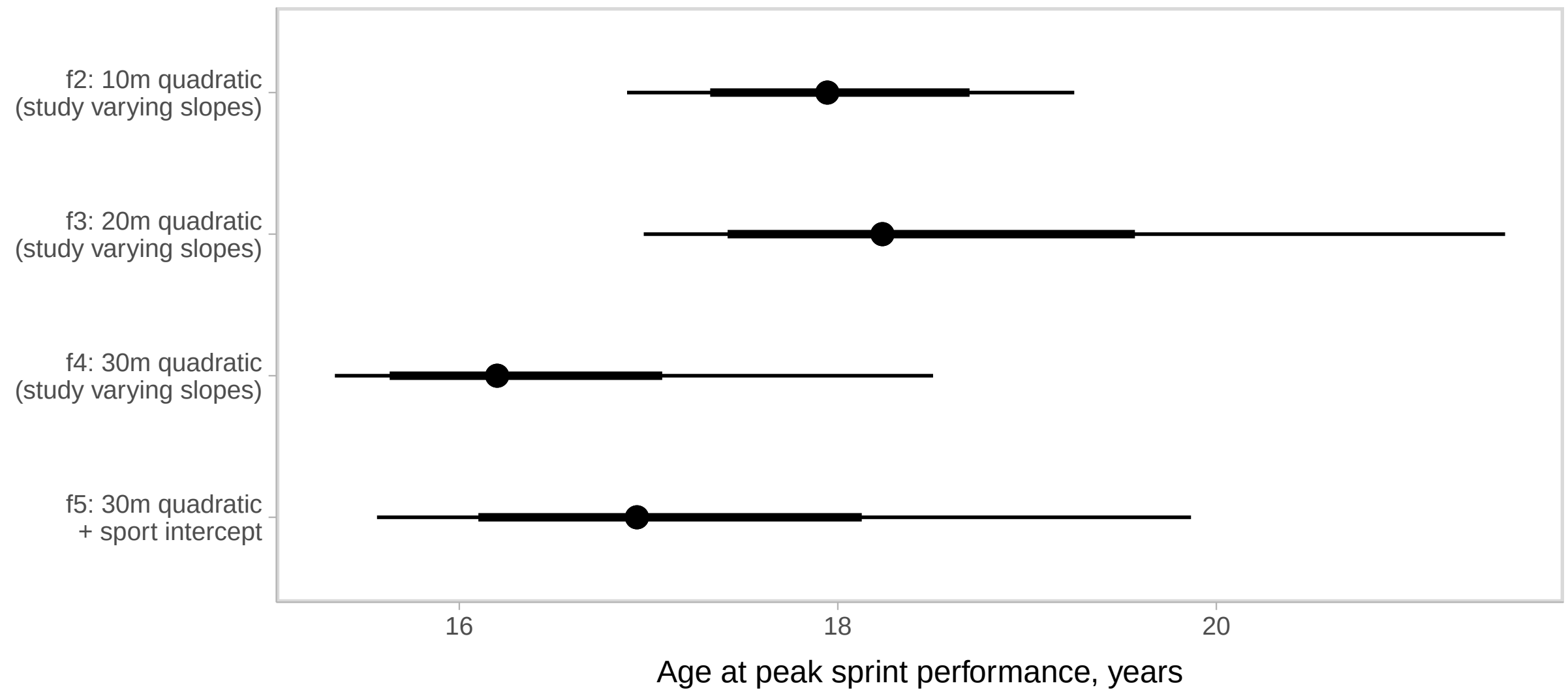

Figure S14: Sensitivity analysis for sprint performance: population-average age at peak sprint performance (minimum sprint time) from quadratic model specifications at three distances. Points indicate posterior means; thick and thin bars show 68% and 90% posterior credible intervals, respectively. The linear 10-m model (f2.1) is omitted as it has no defined vertex.

Supplementary Table S3 presents the APV estimates across all quadratic sprint models. For the 10-m distance, the quadratic model (f2) was compared to the linear model with sport effects (f2.1) via approximate LOO-CV; both models yielded similar predictive performance. For 30-m, the 90% CrIs overlapped between f4 and f5, indicating that sport-level variation did not materially alter the estimated age at peak sprint performance.

Table S3: Sensitivity analysis for sprint performance: population-average age at peak sprint performance (minimum sprint time) with 68% and 90% posterior credible intervals. Model f2.1 (linear, 10m) has no defined vertex and is omitted.

Model	Mean APV (years)	68% CrI	90% CrI
f2: 10m quadratic			
(study varying slopes)	18.00	[17.33, 18.7]	[16.89, 19.25]

Model	Mean APV (years)	68% CrI	90% CrI
f3: 20m quadratic (study varying slopes)	18.08	[17.42, 19.57]	[16.97, 21.53]
f4: 30m quadratic (study varying slopes)	22.70	[15.63, 17.07]	[15.34, 18.5]
f5: 30m quadratic + sport intercept	16.98	[16.1, 18.13]	[15.57, 19.87]

Endurance Performance

Three model specifications were compared:

- fit1 (primary): cubic polynomial; test-type population-level effect (20-m Shuttle Test, Interval Shuttle Run Test, YoYo IR1); uncorrelated varying slopes (intercept, linear, quadratic, cubic) by study.
- fit2 (sensitivity): cubic polynomial; performance-type population-level effect (continuous vs. intermittent exercise); uncorrelated varying slopes by study. Tests whether grouping tests by exercise modality rather than specific protocol changes the estimated APV.
- fit3 (sensitivity): cubic polynomial; test-type population-level effect; uncorrelated varying slopes by study and by sport. Tests whether additional sport-level variation changes the APV.

Supplementary Table S4 presents the APV estimates across all endurance model specifications. The 90% CrIs did not fully overlap across specifications, indicating that the estimated age at peak endurance development velocity was sensitive to one or more modelling choices.

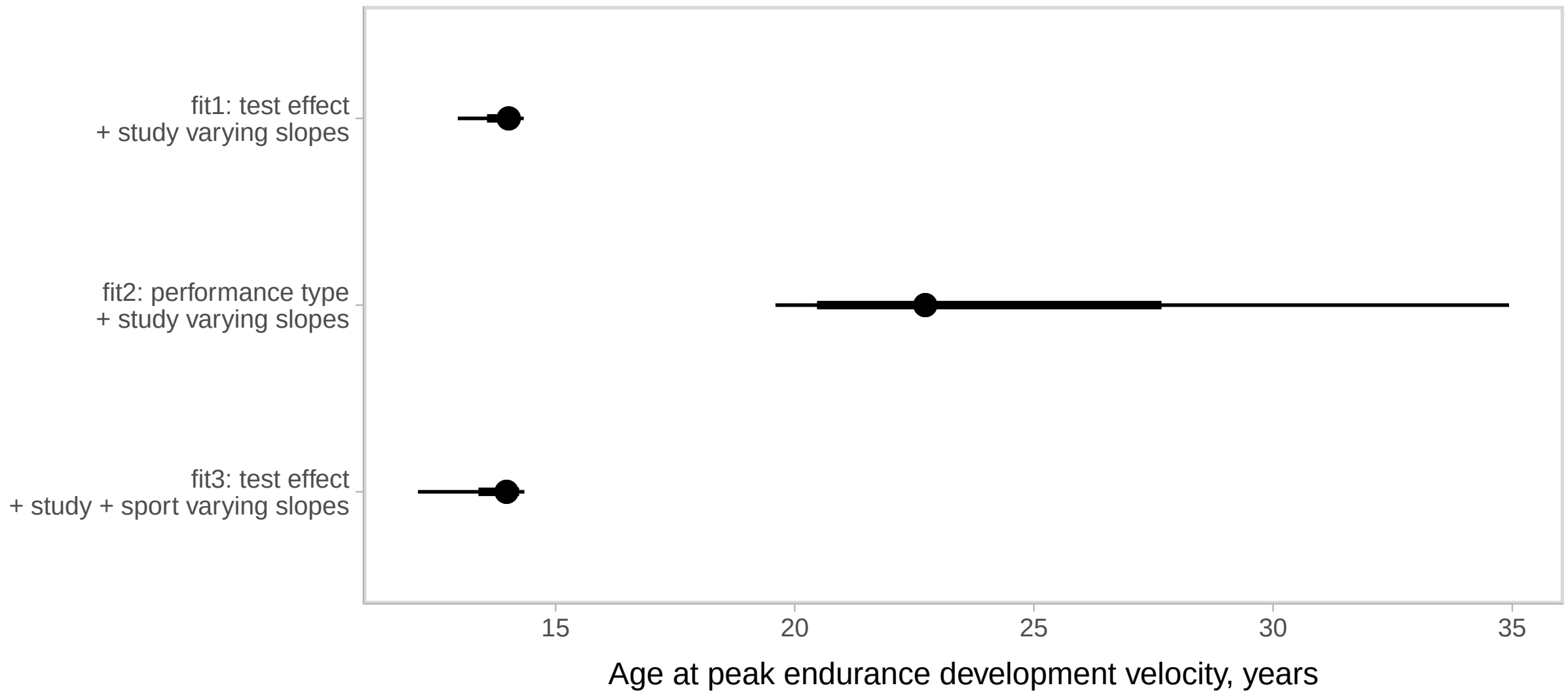

Figure S15: Sensitivity analysis for endurance performance: population-average age at peak endurance development velocity (APV) from three model specifications. Points indicate posterior means; thick and thin bars show 68% and 90% posterior credible intervals, respectively.

Table S4: Sensitivity analysis for endurance performance: population-average age at peak endurance development velocity (APV) with 68% and 90% posterior credible intervals across three model specifications.

Model	Mean APV (years)	68% CrI	90% CrI
fit1: test effect			
+ study varying slopes	13.78	[13.57, 14.25]	[12.95, 14.33]
fit2: performance type			
+ study varying slopes	26.43	[20.46, 27.67]	[19.6, 34.93]
fit3: test effect			
+ study + sport varying slopes	14.22	[13.39, 14.24]	[12.12, 14.35]

Across all three fitness outcomes, sensitivity analyses demonstrated that the estimated ages at peak development velocity were consistent across model specifications differing in group-level effects structure and protocol subset definition, with overlapping 90% posterior CrIs in all comparisons. The qualitative shape of estimated developmental trajectories (non-linear acceleration through early adolescence, peak near mid-adolescence, plateau or deceleration thereafter) was preserved across all

model variants (main text Figures 2–6). These findings support the robustness of the developmental trajectories reported in the main manuscript.

Reference Values

Supplementary Tables S5–S9 present predicted population-level trajectories with 68% CrIs (approximately ±1 posterior SD) at each half-year age increment from 10 to 17 years for vertical jump (S5), 10-m sprint (S6), and endurance performance by protocol (S7–S9). These values are derived from the population-level parameters of the Bayesian multilevel growth curve models and may serve as age-referenced benchmarks for monitoring fitness development in male youth athletes.

Table S5: Reference values for vertical jump (CMJ without arm swing) by age in male youth athletes.

Age	Predicted (cm)	Lower 68% CrI	Upper 68% CrI
10.0	25.5	23.8	27.2
10.5	26.2	24.5	27.8
11.0	27.1	25.5	28.6
11.5	28.1	26.5	29.7
12.0	29.3	27.8	30.8
12.5	30.6	29.1	32.1
13.0	32.0	30.5	33.5
13.5	33.4	31.9	34.9
14.0	34.8	33.4	36.3
14.5	36.2	34.8	37.7
15.0	37.6	36.1	39.0
15.5	38.8	37.3	40.3
16.0	39.9	38.4	41.4
16.5	40.9	39.4	42.4
17.0	41.6	40.0	43.2

Table S6: Reference values for 10-m sprint performance by age in male youth athletes.

Age	Predicted (s)	Lower 68% CrI	Upper 68% CrI
10.0	2.15	2.05	2.25
10.5	2.12	2.02	2.23
11.0	2.10	2.00	2.20
11.5	2.08	1.98	2.18
12.0	2.05	1.95	2.15
12.5	2.03	1.93	2.13
13.0	2.00	1.91	2.10
13.5	1.98	1.89	2.08
14.0	1.96	1.86	2.05
14.5	1.93	1.84	2.03
15.0	1.91	1.82	2.01
15.5	1.89	1.79	1.98
16.0	1.86	1.77	1.96
16.5	1.84	1.74	1.93
17.0	1.81	1.72	1.91

Table S7: Reference values for endurance performance (20m Shuttle Test) by age in male youth athletes.

Age	Predicted (m)	Lower 68% CrI	Upper 68% CrI
10.0	1117	757	1489
10.5	1032	774	1295
11.0	992	812	1173
11.5	993	871	1115
12.0	1027	945	1112
12.5	1087	1027	1151
13.0	1168	1116	1221
13.5	1262	1211	1313
14.0	1363	1314	1413
14.5	1464	1414	1514
15.0	1559	1509	1608
15.5	1641	1590	1690
16.0	1703	1653	1753
16.5	1740	1687	1794
17.0	1744	1677	1810

Table S8: Reference values for endurance performance (Interval Shuttle Run Test) by age in male youth athletes.

Age	Predicted (m)	Lower 68% CrI	Upper 68% CrI
10.0	1229	859	1600
10.5	1143	880	1410
11.0	1104	917	1284
11.5	1105	978	1229
12.0	1139	1051	1227
12.5	1199	1133	1264
13.0	1280	1226	1336
13.5	1374	1323	1427
14.0	1475	1425	1526
14.5	1576	1527	1627
15.0	1671	1621	1722
15.5	1753	1705	1803
16.0	1815	1765	1867
16.5	1852	1798	1906
17.0	1856	1790	1923

Table S9: Reference values for endurance performance (Yoyo IR1) by age in male youth athletes.

Age	Predicted (m)	Lower 68% CrI	Upper 68% CrI
10.0	984	618	1360
10.5	898	635	1163
11.0	859	674	1045
11.5	860	737	981
12.0	893	813	968
12.5	954	901	1001
13.0	1034	994	1072
13.5	1128	1093	1165
14.0	1229	1193	1265
14.5	1331	1296	1367
15.0	1425	1390	1463
15.5	1507	1472	1542
16.0	1570	1534	1606
16.5	1606	1565	1648
17.0	1610	1555	1667
